# A Multi-Level Speed Guidance Cooperative Approach Based on Bidirectional Periodic Green Wave Coordination Under Intelligent and Connected Environment

**DOI:** 10.3390/s25072114

**Published:** 2025-03-27

**Authors:** Luxi Dong, Xiaolan Xie, Lieping Zhang, Shuiwang Li, Zhiqian Yang

**Affiliations:** 1 College of Computer Science and Engineering, Guilin University of Technology, Guilin 541006, China; lishuiwang0721@glut.edu.cn; 2 Guangxi Key Laboratory of Embedded Technology and Intelligent System, Guilin 541004, China; 3 College of Earth Sciences, Guilin University of Technology, Guilin 541004, China; 4 School of Mechanical Engineering, Guilin University of Aerospace Technology, Guilin 541004, China; zlp@guat.edu.cn; 5 College of Arts, Guilin University of Technology, Guilin 541004, China; 2023049@glut.edu.cn

**Keywords:** traffic engineering, connected vehicle environment, sensors, bidirectional periodic green wave, multi-level vehicle speed guidance, collaborative optimization, artificial intelligence

## Abstract

To maximize arterial green wave bandwidth utilization, this study aims to minimize average travel delays at coordinated intersections and maximize vehicle throughput. In view of the aforementioned points, the present paper sets out a collaborative optimization method for the control of related intersection groups. The method combines multi-level speed guidance with green wave coordinated control. In an intelligent and connected environment (ICE), the driving trajectory of the initial vehicle is determined in each optimization cycle following the receipt of active speed guidance. Subsequently, the driving trajectories of subsequent vehicles are calculated, with an assessment made as to whether they can leave the intersection before the end of the green light. The subsequent step involves the calculation of a characteristic index, comprising the average speed of the arterial coordination section and its corresponding phase offset. The phase offset is then optimized with the objective of maximizing the comprehensive bandwidth of green wave coordination within the control range. The maximum average speed and the bidirectional cycle comprehensive green wave bandwidth are employed as the control objectives. Finally, a model is constructed through the combination of multi-level vehicle speed guidance with bidirectional cycle green wave coordinated control. A bi-level combinatorial optimization method is constructed through a combinatorial deep Q learning method, named Deep Q Network-Genetic Algorithm (DQNGA), with the objective of obtaining the global optimal solution. Finally, the reliability of the method is validated using traffic flow data and map sensor data on several associated road sections in a city. The results demonstrate that the proposed method reduces the average delay and number of stops by 20.76% and 44.49%, respectively, outperforming conventional traffic control strategies. This suggests that the issue of inefficient utilization of green light time in arterial coordinated signal control has been effectively addressed. Consequently, the efficiency of intersections in the intelligent and connected environment has been enhanced.

## 1. Introduction

The issue of traffic congestion has become increasingly pertinent in light of the exponential growth in the number of vehicles on the road. The arterial roads that traverse the central districts of urban areas represent a crucial component of the urban traffic system. However, these roads are experiencing an increase in traffic volume, which is the primary cause of traffic congestion. It is therefore essential to enhance the overall traffic capacity of the road network by optimizing the arterial coordination effects, including speed, green ratio, phase offset, green wave bandwidth and other related factors. In the conventional green wave coordinated control approach, the design speed and operational speed of the green wave are frequently fixed, and the green wave bandwidth of arterial roads is not fully exploited. For instance, Sangkey et al. proposed a dynamic bandwidth analysis method utilizing closed-loop signal data [1]. Zhou et al. developed an uneven double cycling method to significantly reduce delay at intersections, thereby providing preliminary guidelines for arterial coordination [2].

When vehicles are in motion on the road, drivers adjust and control their speed by sensing the distances between the front and the following vehicles. This is achieved through a process of visual perception, whereby the driver monitors the distance between their vehicle and the one in front, and adjusts their speed accordingly. This is achieved without the benefit of real-time access to the status of the traffic signals at the corresponding intersection. Consequently, it is difficult to regulate the speed of the vehicle in a timely manner. In recent years, there has been a notable advancement in the sophistication of connected vehicle (CV) technology, which has enabled vehicles to become connected to a multitude of elements, including people, vehicles, infrastructure, and the environment. The travel time of the vehicle is actively adjusted based on information pertaining to the current driving state of the vehicle and the operational state of the roadside traffic signal controller. This is to guarantee that the driver is directed safely through the intersection within the allotted green light time of the current signal cycle. Connected vehicles are able to pass through clusters of associated intersections without stopping in vehicle formations through active speed guidance. Furthermore, enhancing the exchange of information between vehicles and infrastructures can optimize traffic signals to accommodate real-time traffic flows in the future. A number of researchers and institutions have generated substantial achievements in this field to date. This field is divided into the following aspects.

One avenue for future research is the incorporation of a speed guidance model into signal optimization in traditional environments. The traditional study area is primarily concerned with the optimization of signal timing, encompassing the optimization of phase sequence and phase duration for a single intersection [3,4] and multiple intersections (such as road links or two adjacent intersections) [5,6]. In contrast, in traditional environments, the objective of speed guidance is to control speed, resulting in a consistent vehicle speed through variable speed limit facilities, which in turn improves mobility and safety. Liu et al. put forth an integrated model and its solution algorithm for the control of freeway corridors during incidents [7]. Wang et al. developed the model based on the intelligent driver model and variable speed limit control method [8]. However, the aforementioned studies are limited by the technology used to obtain vehicle data. The principal objective of this research is to develop a model for optimizing signal control based on idealized assumptions regarding speed guidance. Nevertheless, this approach does not fully align with the actual operational context.

The second research direction is to investigate the guidance of vehicles at varying speeds in a connected vehicle (CV) environment. This includes the optimization of signal timing at single intersections and the dynamic control and guidance of vehicles at varying speeds on arterial roads. On one hand, in terms of signal timing optimization at a single intersection. Zheng and Liu estimated traffic volumes and then modelled vehicle arrivals at signalized intersections as a time-dependent Poisson process [9]. Sun et al. proposed an innovative intersection operation scheme named as MCross: Maximum Capacity intersection operation scheme with signals [10]. Feng et al. proposed a control framework comprising signal optimization and vehicle trajectory control [11]. Yu et al. presented a mixed integer linear programming model for the purpose of optimizing vehicle trajectories and traffic signals in a connected vehicle environment [12]. Liang et al. used a deep reinforcement learning model for the purpose of controlling the traffic light cycle [13]. Emami et al. adaptively optimized traffic signal plans based on the different penetration information of connected vehicles [14]. Yang et al. proposed a recursive estimation algorithm to update the distribution of the value of times, using the lane choice information of connected vehicles [15]. Rafter et al. proposed a novel algorithm which combines position information from connected vehicles with data obtained from signal timing plans [16]. Mohebifard and Hajbabaie presented a method for cooperative signal timing and traffic metering in urban street networks with various connected vehicle market penetration rates [17]. On the other hand, in the aspects of dynamic speed control and guidance in arterial roads, Kamal et al. presented a novel control system to drive a vehicle efficiently on roads containing varying traffic and signals at intersections [18]. Wu et al. developed two vehicle speed guidance methods to decrease the delay and number of stops at intersections [19]. Lin et al. provided an algorithm to obtain vehicles’ dynamics parameters in a variant-speed area [20]. Ma et al. presented a partition-enabled multi-mode band model that is designed to solve the signal coordination problem [21]. Xu et al. optimized the traffic signal timing and vehicles’ speed trajectories at an isolated intersection [22]. Wu et al. proposed a novel joint control method based on model predictive control and connected vehicles for on-ramp metering and speed guidance on the urban expressway [23]. Zhou et al. proposed a hybrid cooperative intersection control framework consisting of microscopic-level virtual platooning control and macroscopic-level traffic flow regulation with connected vehicles [24]. Chen et al. built three conflict modes of different traffic movements and presented safe speed-dependent constraints for them [25]. Liang et al. proposed a decentralized signal control algorithm that leverages connected vehicles’ information to improve urban traffic operations [26]. Chen et al. developed an adaptive signal control that adjusts the signal timing plan while considering both the urban adjacent intersections’ traffic volume and the vehicles’ waiting time [27]. Yang et al. proposed a novel approach to integrate optimal control of perimeter intersections into a perimeter control scheme [28].

As demonstrated in the literature review, speed guidance and signal control remain prominent research topics. When optimizing signal timing at a single intersection, it is possible to jointly optimize signal timing parameters and vehicle speed in order to facilitate more accurate vehicle arrival planning. Nevertheless, previous studies have proposed that the trajectory of each vehicle should be determined based on fixed signal timing and an ideal green wave bandwidth. However, existing methods fail to address communication reliability and multi-lane traffic dynamics, limiting their practical applicability. Furthermore, the extant signal timing optimization method fails to consider the actual dynamics of vehicles in order to regulate their movements. A second limitation of the current study is that it only examined simple intersections or arterials with only one lane per approach. However, it should be noted that multi-lane approaches to intersections are a common feature of urban road networks. It is therefore necessary to develop a control method that can accommodate the general case, including groups of intersections with multi-lane approaches and varying traffic states. Thirdly, control strategies based on limited information may result in suboptimal operations. The absence of global traffic information may result in collaborative control optimization strategies that deviate from the optimal solution for the system as a whole.

Another avenue for investigation is the optimization of connected vehicle trajectories with the objective of reducing fuel and energy consumption, as well as phase offset, given fixed signal timings. Naturally, the optimal control methods were modelled based on the vehicle speeds or acceleration rates [29,30,31]. Liu et al. developed an optimal mode selection and resource allocation (MSRA) policy that maximizes the long-term overall throughput of a time-varying dynamic energy harvesting D2D-enabled cellular network (EH-DCN), subject to an age of information (AoI) constraint [32]. He et al. proposed a multi-stage optimal control formulation [33]. Tang et al. introduced a speed guidance strategy into a car-following model to analyze the relationship between driving behavior and fuel consumption [34]. Zhao and Zhang employed an online learning-based driving dynamics prediction model to forecast a set of uncertain driving states of the preceding vehicle [35]. He et al. proposed an overall control strategy for heterogeneous vehicle platoons with the objective of minimizing energy consumption [36]. Recent studies have proposed the integration of traffic signal control and vehicle trajectory control within a unified framework. Wu et al. proposed an integrated signal coordination control model to optimize the dynamic travel speed and signal offsets [37]. Tajalli et al. presented distributed optimization and coordination algorithms for dynamic speed optimization of connected and autonomous vehicles [38]. Wang et al. developed a joint control model for optimizing the connected vehicle speeds and coordinating signals along an urban arterial simultaneously [39]. Bie et al. proposed a dynamic headway control method for a high-frequency route with a bus lane [40]. Liu et al. developed a reservation-based cooperative transit signal priority mechanism system to optimize the signal scheme and speed guidance [41]. In view of the non-convexity of formulated problems with a large number of optimization variables and the dynamics of EH-DCNs with high real-time requirements, it is challenging to obtain a long-term optimal MSRA policy through conventional optimization methods. Fortunately, deep reinforcement learning (DRL), often represented by deep Q-networks (DQN) and deep deterministic policy gradient (DDPG), is a powerful tool for achieving optimal solutions in time-varying dynamic EH-DCNs. However, it should be noted that DQN demonstrates notable proficiency in addressing discrete variable problems but encounters challenges in the context of problems incorporating continuous variables. While DDPG is capable of handling problems involving both continuous and discrete variables, the issue of Q-value overestimation persists and requires resolution. To address this challenge, we have developed the MSRA twin delayed deep deterministic policy gradient (MSRA-TD3) algorithm, with the objective of enhancing the efficacy of DDPG in dealing with problems that involve both continuous and discrete variables.

In summary, the majority of the aforementioned studies have employed fixed signal timings for traffic signals, with control strategies designed to optimize energy consumption at specific speeds. Nevertheless, existing studies have indicated that platooning may not be able to maintain robust stability at specific speeds, which could potentially affect the operational efficiency of these methods. The microscopic nature of the existing algorithms, in which the trajectory of each connected vehicle is controlled, leads to a significant increase in the complexity of the algorithms used to solve the problem. Therefore, there is a clear need to improve the aforementioned algorithms and optimize their response efficiency.

In general, several problems remain to be addressed in the current research.

(1) Despite the existence of some studies investigating vehicle speed guidance, the majority of current research focuses on a single intersection or the traditional arterial green wave coordinated control. There has been little consideration of the demand for additional green waves, which is the unused portion of the green wave under arterial green wave coordinated control. The introduction of an additional green wave has some impact on vehicle speed, which in turn has an impact on phase offset.
(2) In the context of traditional traffic control, it is not uncommon for vehicles to experience difficulties in maintaining a constant speed, which can lead to a stop-and-go situation. To address this problem, it has been proposed to implement speed guidance for individual vehicles rather than for groups. However, further improvements to the control system are needed. In the context of coordinated control models, the parameters typically used for green wave optimization include fixed elements such as travel path, initial queue length, driving safety and driving speed. It is important to recognize that these models are not global and not fully dynamic optimization models.
(3) The connected vehicle fleets are intercepted at the upstream intersection as they move in multiple directions across different lanes, in accordance with the green wave band speed designed for bidirectional cycle-coordinated intersection groups. The effect of the green wave is somewhat limited. There is a lack of research investigating the correlation and trade-off analysis between the continuous passing speed of connected vehicles and the safe and accurate multi-level speed active guidance in a bidirectional cycle-coordinated green wave control environment.

In summary, existing methods have three limitations: (1) static green wave designs that ignore dynamic traffic variations; (2) underutilization of additional green wave bandwidth; and (3) absence of joint vehicle–signal optimization. This paper addresses these gaps by proposing a bidirectional cycle green wave coordination framework integrated with multi-level speed guidance, enabled by a novel DQNGA hybrid algorithm for global optimization. Thus, this work contributes to the field in two key aspects: Firstly, a bidirectional cycle green wave coordinated control and multi-level vehicle speed guidance collaborative optimization model based on multi-dimensional comprehensive control objectives under a connected vehicle environment is established. Secondly, the model employs an artificial intelligence algorithm that is capable of achieving global optimization. This collaborative optimization model is distinguished from other studies by its utilization of the arterial green wave bandwidth time, including additional green waves, and its ability to adaptively determine the global optimal speed based on the real-time traffic signal state. This enables connected vehicle fleets to pass through arterial-related intersections at the global optimal speed without stopping.

The remainder of this paper is organized into the following sections: Section 2 provides a detailed description of the problem. Section 3 outlines the methodology and mathematical modelling employed. Section 4 presents a case study. The conclusion is presented in Section 5.

## 2. Problem Description

**Figure 1:**
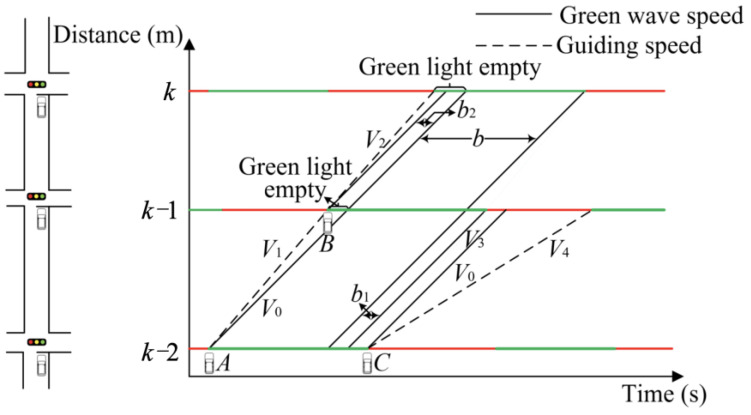
Schematic diagram of the practical arterial green wave coordinated control model.

The model for coordinating the ideal arterial green wave is constructed based on the assumption of stable traffic flow [42,43]. In a road section where the green wave coordinated control is in operation, vehicles are permitted to travel at the green wave speed to achieve non-stop travel through the intersection. Nevertheless, the practical application of the green wave coordinated control model is presented in Figure 1 (for further details, please refer to Figure 1, which presents the control model of upstream coordination direction). It is generally acknowledged that the coordination cycle cannot be guaranteed to be consistent. Furthermore, the phase offsets between some intersections and their upstream and downstream counterparts are also inconsistent, which may result in the generation of additional green waves. Figure 1 (see *b*_1_ and *b*_2_) illustrates that vehicles passing within the public green wave bandwidth *b* continue to travel at the green wave speed (defined as *v*_0_) and can pass through the green wave smoothly. As shown in Figure 1, vehicles A and B are travelling at the green wave speed, which results in an empty green light at the downstream intersection. The issue can be resolved by instructing vehicles A and B to accelerate to speeds *V*_1_ and *V*_2_, respectively. In contrast, Vehicle C is also travelling at the green wave speed but encounters a red light at the downstream intersection, resulting in traffic delays. If vehicle C accelerates to a speed of *V*_3_, it may pass the intersection during the current green light cycle. An alternative option would be for the vehicle to decelerate to a speed of *V*_4_ in order to pass the intersection during the next green light cycle.

In an intelligent and connected environment, connected vehicles have the capacity to disseminate operating information (for example, speed, lane changing, acceleration, position) to other vehicles, while concurrently detecting the real-time traffic state at intersections (for example, traffic flow, traffic capacity, travel time) and traffic signal timing state (for example, red light time, green light time, cycle length) via vehicle-to-infrastructure (V2I). Additionally, the connected vehicles receive information regarding traffic signals and guidance speeds. However, the inherent randomness of vehicle speeds and queue lengths poses significant challenges in practical implementation. For instance, the green light time in other phases must be reduced to adapt to the arterial coordinated control.

**Figure 2:**
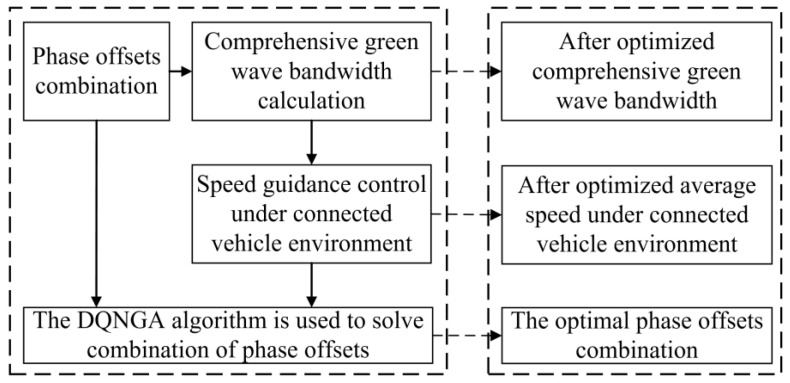
Diagram of optimization model.

In contrast to the previous method, the acquisition of real-time traffic data enables the implementation of bidirectional green wave coordinated control through active speed guidance in a connected vehicle environment. The system is capable of self-adjusting in real time, enabling the continuous flow of vehicles through intersection groups without stopping. In addition, the model is able to achieve the optimum comprehensive green wave bandwidth for intersection groups associated with arterial roads while adapting to vehicle operating states. This has the potential to increase the average speed of connected vehicle fleets traversing arterial roads, as well as optimizing the capacity and traffic service of said roads through a combination of optimal and self-adaptive phase offsets. The overall concept is illustrated in Figure 2.

## 3. Mathematical Modeling

The paper presents a collaborative optimization method that integrates fleet motion control, speed guidance and green wave bandwidth optimization with the aim of improving the traffic capacity of arterial-related intersection groups. Phase offset optimization is based on real-time traffic flow, with all connected vehicles maintaining a fleet and operating at an optimal average speed. Ultimately, the goal is to facilitate the uninterrupted passage of as many connected vehicles as possible through arterial-related intersection groups.

### 3.1. Model Assumptions and Parameter Definitions

The model is established on the following assumptions:

(1) Vehicles within the control area are assumed to fully comply with speed guidance instructions [44].
(2) Vehicles will not change lanes after entering the control area [45].
(3) The interference of pedestrians and non-motorized vehicles is not considered [46].
(4) Connected vehicles can achieve mutual cooperative control when they are in good communication with each other and with the roadside unit. The central control unit is capable of executing the assigned driving tasks [47].
(5) The on-board units of connected vehicles and roadside units communicate with each other via the IEEE 802.11p protocol, which ensures the accuracy and real-time performance of the collected information [48].

Table 1 presents the definitions of the principal parameters of the optimization model.

### 3.2. Bidirectional Cycle Green Wave Coordinated Control Based on Multi-Level Speed Guidance Collaborative Method Under a Connected Vehicle Environment

#### 3.2.1. Vehicle Speed Guidance Strategy Under a Connected Vehicle Environment

**Figure 3:**
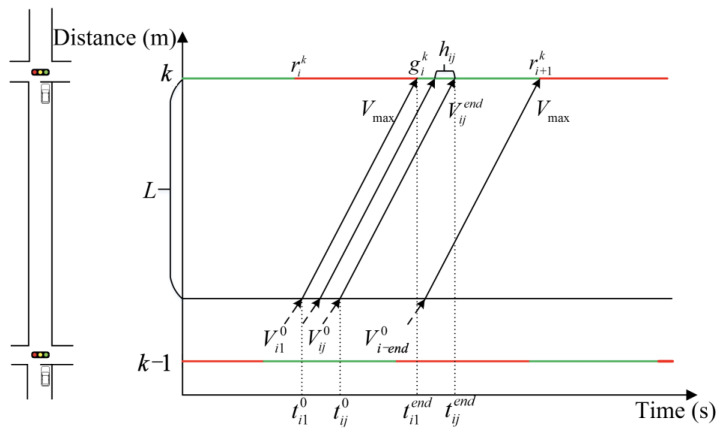
Schematic diagram of multi-level speed guidance target state.

In a connected vehicle environment, a vehicle transmits information about its position and speed to the central control unit when it enters the defined control area. The size of the control area depends on the effective distance between the vehicle and the infrastructure. The objective of the optimization process is to determine the initial and target states of the vehicles. In order to reduce vehicle travel time and optimize the use of green wave bandwidth to increase vehicle throughput, it is necessary to allow vehicles to pass through arterial intersection groups at the maximum speed permitted by the road. This ensures that all vehicles reach the same target state before reaching the downstream intersection. This is achieved by the multi-level speed control method proposed in this paper. Figure 3 schematically illustrates the optimal speed control curve.

The target state model is presented in Equations (1) and (2):

(1) $$\left\{\begin{array}{c} V_{ij}^{end}=V_{\max}\\ t_{ij}^{end}=t_{i1}^{end}+\sum_{n=2}^{j}h_{in}\\ l_{ij}^{end}=l_{i1}^{end} \end{array}\right.$$

(2) $$h_{ij}\geq \frac{S_{0}+len_{i\left(j-1\right)}}{V_{ij}^{end}}\geq h_{t}$$

where *r^k^_i_* and *r^k^_i_*_+1_ represent the start time of the red light at the *i*th optimization cycle and the *i* + 1th optimization cycle of intersection *k*, respectively. *g^k^_i_* represents the start time of the green light at the *i*th optimization cycle of intersection *k*. In this paper, we take the time from the start of the red light to the end of the green light at the downstream intersection as one optimization cycle.

In the context of speed guidance, it is of paramount importance to consider both vehicle acceleration and deceleration. Nevertheless, numerous studies tend to neglect the transitional stage between acceleration and deceleration. The objective of this paper is to reduce non-uniform motion time by focusing on the maximum acceleration (referred to as *a*_max_) with both positive and negative values. Upon entering the control area, the vehicle must undergo uniform acceleration until it reaches a velocity of *V*_max_. The optimization process comprises at least two stages. If the vehicle cannot pass through the intersection during the current green light, it is imperative that it is guided to maintain its speed in order to avoid the necessity of stopping and queuing at the intersection. This will permit the vehicle to pass through the intersection during the next green light. To ensure that the vehicle does not stop and queue, it will pass through a suitable uniform speed transitional stage before accelerating to *V*_max_. The speed control process of the vehicle will be divided into four stages. The first stage involves the vehicle accelerating or decelerating to reach the first guiding speed. The second stage is the uniform motion stage at the first guiding speed. The third stage comprises the second uniform acceleration motion, which accelerates to *V*_max_. The fourth stage involves uniform motion with *V*_max_. The subsequent paragraph will provide a detailed discussion of the two-stage and four-stage methods.

The multi-level speed guidance control method for each optimization cycle is described as follows:

Step 1: Determine the speed control curve for the first vehicle in each optimization cycle.

The leading vehicle is defined as the first vehicle to pass through the intersection after the start of each optimization cycle. By determining the speed control curve of the leading vehicle, it is possible to calculate the time range for subsequent vehicles to reach the downstream intersection and to establish the basic line shape of the speed control curve for those vehicles.

A speed control curve is calculated for the leading vehicle based on its initial speed upon arrival at the control area, the distance between the vehicle and the downstream intersection, and the signal state information of the downstream intersection. The speed control curve for the leading vehicle may be constituted by either two-stage speed control curves or four-stage speed control curves. The optimization method is given as follows.

(a) Two-stage method

**Figure 4:**
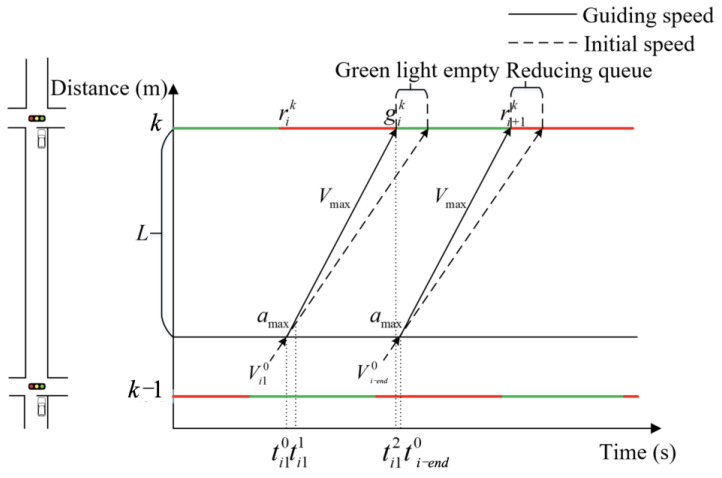
Schematic diagram of speed guidance two-stage method.

When entering the control area with the intention of passing the intersection smoothly, the leading vehicle is permitted to accelerate directly to *V*_max_. This not only addresses the issue of green light-empty release of additional green wave bandwidth but also reduces the queue and improves the traffic efficiency of intersection groups. Figure 4 shows the schematic diagram of active speed guidance in the two-stage method.

The time range for the leading vehicle to enter the control area applicable to the two-stage method can be calculated by determining the start and end times of the green light at the downstream intersection. When the leading vehicle arrives at the intersection exactly at the start time of the green light, the time (defined as $t_{i1}^{0}$) at which the vehicle enters the control area can be calculated by Equation (3). In order to address this theoretical optimization, the following proposal is put forward: Probabilistic Compliance Models: The introduction of partial adherence rates (defined as *η*_compliance_).

(3) $$\left\{\begin{array}{l} V_{max}=V_{i1}^{0}+a_{max}(t_{i1}^{1}-t_{i1}^{0})\\ l_{1}=\frac{{\left(V_{max}\right)}^{2}-{\left(V_{i1}^{0}\right)}^{2}}{2a_{max}}\\ l_{2}=V_{max}\left(t_{i1}^{2}-t_{i1}^{1}\right)\\ L=l_{1}+l_{2}\\ \eta_{compliance}=0.8 \end{array}\right.$$

Derive $t_{i1}^{0}$ from Equation (3). The result is shown in Equation (4).

(4) $$\left\{\begin{array}{c} t_{i1}^{0}=t_{i1}^{2}-\frac{L}{V_{\max}}+\frac{{\left(V_{\max}\right)}^{2}-{\left(V_{i1}^{0}\right)}^{2}}{2a_{\max}V_{\max}}-\frac{V_{\max}-V_{i1}^{0}}{a_{\max}}\\ t_{i1}^{1}=t_{i1}^{2}-\frac{L}{V_{\max}}+\frac{{\left(V_{\max}\right)}^{2}-{\left(V_{i1}^{0}\right)}^{2}}{2a_{\max}V_{\max}} \end{array}\right.$$

Similarly, as demonstrated in Equation (5), it is possible to calculate the time (defined as $t_{i-end}^{0}$) when the leading vehicle enters the control area and arrives at the intersection precisely at the end of the green light time.

(5) $$t_{i-end}^{0}=t_{i-end}^{2}-\frac{L}{V_{\max}}+\frac{{\left(V_{\max}\right)}^{2}-{\left(V_{i-end}^{0}\right)}^{2}}{2a_{\max}V_{\max}}-\frac{V_{\max}-V_{i-end}^{0}}{a_{\max}}$$

Thus, the two-stage speed guidance control method is applicable to the leading vehicle in the $\left[t_{i1}^{0},t_{i-end}^{0}\right]$.

(b) Four-stage method

**Figure 5:**
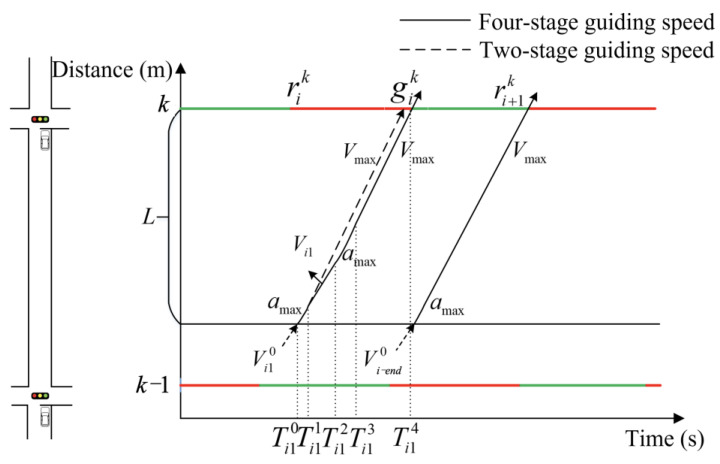
Schematic diagram of speed guidance four-stage method.

The two-stage method calculations indicate that the leading vehicle entering the control area will encounter a red-light queue at the downstream intersection. In order to reduce the average delay time and ensure that the vehicle reaches *V*_max_ before entering the intersection, it is necessary to employ the four-stage method in order to induce the vehicle to pass through the intersection without stopping. The diagram of speed guidance is shown in Figure 5.

The objective of this paper is to identify the optimal speed control curve for the vehicle, which should result in the minimum time required for uniform speed change and the maximum guiding vehicle speed. The four-stage speed control curve displays a relatively high degree of flexibility. The optimal speed control curve is calculated as follows.

The time range for the leading vehicle to enter the control area applicable to the four-stage method can be calculated by determining the start and end times of the green light at the downstream intersection. When the leading vehicle arrives at the intersection exactly at the start time of the green light, the time (defined as $T_{i1}^{0}$) at which the vehicle enters the control area can be calculated by Equations (6) and (7). As shown in Figure 5, Equation (6) can be obtained from the relationship between speed and acceleration.

(6) $$\left\{\begin{array}{c} V_{i1}=V_{i1}^{0}+a_{max}(T_{i1}^{1}-T_{i1}^{0})\\ V_{max}=V_{i1}+a_{max}(T_{i1}^{3}-T_{i1}^{2}) \end{array}\right.$$

The total displacement within the control range (defined as *L*) is measured when the vehicle is in the four-phase method, which can be calculated by Equation (7).

(7) $$\left\{\begin{array}{l} L_{1}=\frac{{\left(V_{i1}\right)}^{2}-{\left(V_{i1}^{0}\right)}^{2}}{2a_{max}}\\ L_{2}=V_{i1}\left(T_{i1}^{2}-T_{i1}^{1}\right)\\ L_{3}=\frac{{\left(V_{max}\right)}^{2}-{\left(V_{i1}\right)}^{2}}{2a_{max}}\\ L_{4}=V_{max}\left(T_{i1}^{4}-T_{i1}^{3}\right)\\ L=L_{1}+L_{2}+L_{3}+L_{4} \end{array}\right.$$

Similarly, from Figure 5, the time relationship for each stage in the four-stage method is shown in Equation (8)

(8) $$\left\{\begin{array}{l} T_{ij}^{1}-T_{ij}^{0}=T_{1}\\ T_{ij}^{2}-T_{ij}^{1}=T_{2}\\ T_{ij}^{3}-T_{ij}^{2}=T_{3}\\ T_{ij}^{4}-T_{ij}^{3}=T_{4}\\ T_{ij}^{4}-T_{ij}^{0}=T \end{array}\right.$$

The *T*_3_ and *V_i_*_1_ are calculated by Equations (6)–(8), respectively, as shown in Equation (9).

(9) $$\left\{\begin{array}{c} T_{3}=\frac{V_{\max}-V_{i1}^{0}-a_{\max}T_{1}}{a_{\max}}\\ V_{i1}=V_{i1}^{0}+a_{\max}T_{1} \end{array}\right.$$

The objective function is calculated in Equation (10).

(10) $$\min F=\frac{T_{1}}{T}+\frac{V_{\max}-V_{i1}^{0}-a_{\max}T_{1}}{a_{\max}T}+\frac{V_{\max}}{V_{i1}^{0}+a_{\max}T}$$

The appropriate *T*_1_ is calculated by Equation (10), after which the speed control curve of the leading vehicle is determined.

Step 2: Calculate the speed control curve of subsequent vehicles.

The speed control curve of the subsequent vehicles can be computed sequentially, following the determination of the speed control curve of the leading vehicle. The ensuing discussion will address these two cases in turn, with reference to the aforementioned leading vehicle.

(a) Two-stage method

If the leading vehicle is able to successfully negotiate the intersection in a manner that is both smooth and efficient by employing the two-stage method, it is of the utmost importance that subsequent vehicles in the optimization cycle are also capable of utilizing this method. The aforementioned calculations demonstrate that the two-stage method is applied to subsequent vehicles entering the control area within the interval of $\left[t_{i1}^{0},t_{i-end}^{0}\right]$.

(b) Four-stage method

When the leading vehicle is able to pass through the intersection in a smooth and uninterrupted manner via the four-stage method, subsequent vehicles do not adopt this method. Upon entering the control area, the speed control curves for both the two-stage and four-stage methods are calculated simultaneously. The speed control calculation method for subsequent vehicles is similar to that of the leading vehicle. However, in contrast to the leading vehicle, subsequent vehicles are required to maintain a safe driving distance from the vehicle in front while travelling. This requirement is satisfied by Equation (11).

(11) $$\Delta S\left(t\right)=S_{i\left(j-1\right)}\left(t\right)-S_{ij}\left(t\right)\geq S$$

If subsequent vehicles can pass through the intersection smoothly via the two-stage method, then the method will be adopted. Otherwise, the four-stage method will be used.

Step 3: Determine whether the vehicle can leave the intersection before the green light ends.

**Figure 6:**
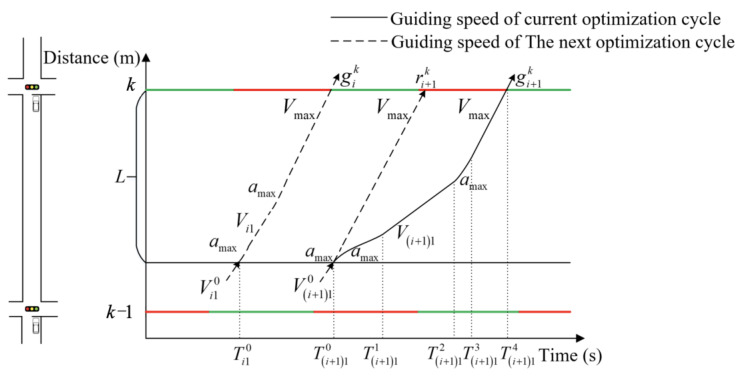
Schematic diagram of speed guidance without stopping.

Upon entering the control area, calculations demonstrate that the vehicle will encounter a red-light queue upon reaching the downstream intersection. To minimize average delay time and ensure the vehicle reaches *V*_max_ before entering the intersection, it is necessary to guide the vehicle to pass through the intersection without stopping in the next optimization cycle. This will result in the vehicle becoming the leading vehicle in the subsequent optimization cycle, as shown in Figure 6.

Step 4: Calculate the traffic shockwave under a connected vehicle environment.

Step 4.1: Calculate the traffic gathering wave.

When the traffic signal turns red, the leading vehicle begins to stop, and subsequent vehicles adjust their speed and trajectory in accordance with the leading vehicle. The vehicle establishes a communication connection with the roadside unit and on-board unit. At this point, the on-board unit (OBU) transmits a continuous stream of requests to the roadside unit (RSU) for the real-time intersection group signal timing schemes and the remaining red light time of the current phase. This paper assumes a communication delay (defined as *t_delay_*) between vehicles and infrastructure in a connected vehicle environment.

The time for the gathering wave to travel to the *j*th vehicle (defined as *T_j_*__*gather*_) can be calculated from Equation (12).

(12) $$T_{j\_gather}=\sigma \times \frac{e^{-\varsigma }-e^{-\varsigma j}}{\varsigma }\times \frac{k_{2}-k_{1}}{k_{1}k_{2}V_{ij}}$$

where ς and σ are constants to be calibrated based on the traffic data. *k*_1_ is the normal travel speed of the traffic flow. *k*_2_ is the congestion density of the traffic flow.

Step 4.2: Calculate the traffic dissipation wave.

When the traffic signal turns green, vehicles that were previously stopped in front of the stop line begin to move and accelerate through the intersection. The speed of the wave begins to travel backwards, following the dissipation wave received by the vehicle after time *t_delay_*__1_. The vehicle accelerates quickly until it reaches its maximum speed and then passes through the intersection at a constant speed. The time it takes for the dissipation wave to reach the *j*th vehicle at this stage (defined as *T_j_*__*dissipat*_) can be calculated using Equation (13).

(13) $$T_{j\_dissipat}=\sigma \times \frac{e^{-\varsigma }-e^{-\varsigma j}}{\varsigma }\times h_{t}-\frac{1}{k_{2}V_{\max}}$$

Equation (14) calculates the red-light time for *I*th phase (including the phases in all four directions of each intersection, such as the eastward, westward, southward, and northward phases).

(14) $$R_{t}=C-L_{I}-g_{I}$$

where *g_I_* represents the effective green light time at *I*th phase. *C* is the cycle time. *L_I_* is the loss time at phase *I* of the intersection. The queue time for the signal control scheme at *I*th phase is *T_j_*__*n*_, which is calculated by Equation (15).

(15) $$T_{j\_n}=(R_{t}-t_{delay})-T_{j\_gather}+T_{j\_dissipat}$$

Substituting Equations (13)–(15) into Equation (16),

(16) $$T_{j\_n}=\left[C-L_{I}-g_{I}-t_{delay}\right]-\sigma \times \frac{e^{-\varsigma }-e^{-\varsigma j}}{\varsigma }\times \left(\frac{k_{2}-k_{1}}{k_{1}k_{2}V_{ij}}-h_{t}+\frac{1}{k_{2}V_{\max}}\right)$$

Step 5: Calculate an evaluation index for combination of the green wave and the turn wave.

The traffic state within the green wave is quantified through the measurement of the speed and trajectory of connected vehicles, with the vehicle origin–destination (OD) trip matrix subsequently recorded.

The variables *m* and *k* represent traffic flow (including going straight and turning right or left) into and away from the green wave section ID (i.e., 1 < *m* < *n*, 1 < *k* < *n* + 1), respectively. As the arterial green wave is bidirectional, *m* ≠ *k*. *N_m_*_,*k*_ represents the number of vehicles travelling from section *m* to section *k*, whether stopping or continuing their journey (including the turn wave).

Define the vehicle origin–destination (OD) matrix. When *m* − *k* < 2, the vehicle is not entering the arterial green wave of coordinated vehicles.

When *m* − *k* ≥ 2 is satisfied, the vehicle is a vehicle that enters the arterial green wave coordination. Additionally, count the number of vehicles entering the road section where the green wave is located from the section *m*.

(17) $$N_{m}=\sum_{m=k}^{n+1}N_{m,k}$$

The evaluation index using the number of vehicles OD trip matrix is calculated as follows.

Step 5.1: Design the actual traffic efficiency value for the green wave.

The efficiency of the green wave coordination is measured by the actual traffic efficiency value, defined as *I_R_*. This value represents the number of intersections that a vehicle (including going straight and turning right or left) passes through continuously while in the green wave coordination and control scheme. The equation for calculating *I_R_* for all vehicles is based on the product of the number of vehicles and the number of intersections they pass through. It is crucial to emphasize that the scoring value for a single vehicle, *I_R_*, represents the objective scoring for the vehicle in the arterial green wave in its actual operational state. The equation for calculating *I_R_* for all vehicles is as follows:

(18) $$I_{R}=\sum_{m=1}^{n-1}\sum_{k=1}^{n+1}\left[N_{m,k+2}\times (m-k+2)\right]$$

Step 5.2: Design the ideal value for green wave traffic efficiency.

In order to enter the green wave, it is optimal for vehicles (including those travelling straight and turning right or left) to depart from the first encountered green light intersection and drive away from the arterial green wave through the various intersections that have green lights. The optimal number of consecutive intersections through the green wave (*I_l_*) can be determined by combining it with the vehicle OD trip matrix. This represents the green wave control effect of the ideal state of operation. The product of the departure point of all OD trips is multiplied by *I_l_* to obtain the objective scoring value of the arterial green wave for the ideal number of consecutive intersections through. For all vehicles, the value of *I_l_* is calculated using Equation (19).

(19) $$I_{l}=\sum_{m=1}^{n-1}\left[N_{m}\times (n+1-m)\right]$$

Step 5.3: Design the disturbance value for green wave traffic efficiency.

The calculation of the optimal traffic efficiency value for the green wave excludes vehicles that do not enter the arterial green wave coordination from the statistical range. This guarantees that the evaluation outcomes of the vehicle coordination are consistent, even in circumstances where there are a considerable number of vehicles that do not adhere to the arterial green wave coordination. It is imperative to circumvent such scenarios to ensure the integrity of the study’s findings. In addition, it is acknowledged that different green wave coordination schemes possess control scenarios that are not conducive to repetition in practical applications. In order to ensure the objectivity of the evaluation results, the definition of the green wave incorporates a perturbation value. This value is of particular significance in coordinating the green wave on road sections with varying traffic volumes and intersection patterns. The objective scoring methodology considers only vehicles that are part of the green wave coordination, represented by a single vehicle (defined as *I_D_*). The scoring value for vehicles not included in the green wave coordination is defined as 1. The equation for all vehicle *I_D_* is calculated as follows:

(20) $$I_{D}=\sum_{m=1}^{n}N_{m,k}\times 1+\sum_{m=1}^{n+1}N_{m,k+1}\times 1$$

Step 5.4: Design the evaluation index for green wave coordination.

The evaluation of green wave coordination is based on the actual traffic efficiency value. The size of the arterial green wave disturbance value is taken into consideration to calculate the degree of approximation of the value in question in comparison to the ideal operating state of the green wave. The evaluation index (defined as *I_E_*) for green wave coordination is calculated using Equation (21).

(21) $$I_{E}=\frac{I_{R}}{I_{l}-I_{D}}$$

The traffic state of the arterial green wave is defined as *S*, and the traffic state of the ideal green wave is defined as *S_I_*. When the traffic state of the arterial green wave is approaching the ideal condition, the impact of disturbances on the green wave is negligible ($\underset{S\to S_{I}}{\lim}I_{R}=0$), and the actual efficiency value (*I_D_*) approaches the ideal efficiency value (*I_l_*) ($\underset{S\to S_{I}}{\lim}I_{D}=I_{I}$). Therefore, the maximum value of the green wave coordinated evaluation index represents the ideal state ($\underset{S\to S_{I}}{\lim}I_{E}=\underset{S\to S_{I}}{\lim}\frac{I_{R}}{I_{I}-I_{D}}=\underset{S\to S_{I}}{\lim}\frac{I_{I}}{I_{I}}=1$).

To evaluate the effectiveness of bidirectional green wave coordinated controls, we use the evaluation index of green wave coordination (*I_E_*). The higher the value of *I_E_*, the better the green wave control. If $\left|I_{E1}-I_{E2}\right|\leq \varepsilon$ (where *ε* is any infinitesimal), the value of *I_R_* is compared. The higher the value of *I_R_*, the better the green wave control. If $\left|I_{R1}-I_{R2}\right|\leq \varepsilon$, the value of *I_D_* is further compared. The higher the value of *I_D_*, the better the effect of the green wave control.

#### 3.2.2. Constructing the Collaborative Model Under a Connected Vehicle Environment

(a) *Correlation analysis of speed, signal offset and green wave bandwidth*

In a connected vehicle environment, real-time information about the vehicles is obtained, including speed, position, vehicle spacing, and distance. Subsequently, the average speed of each pertinent roadway is determined through the application of Equation (22) following the completion of data pre-processing.

(22) $$\begin{array}{l} \bar{V}=\sum_{j=1}^{n}\frac{v_{j}}{n}\\ s.t.\;\left\{\begin{array}{l} V_{ij}^{end}=V_{\max}\\ t_{ij}^{end}=t_{i1}^{end}+\sum_{n=2}^{j}h_{in}\\ l_{ij}^{end}=l_{i1}^{end}\\ h_{ij}\geq \frac{S_{0}+len_{i\left(j-1\right)}}{V_{ij}^{end}}\geq h_{t}\\ v_{i}(t)\leq V_{\max},\;t\in \left[t_{i1}^{0},t_{i-end}^{0}\right]\;\\ v_{i}(t)\geq V_{\min},\;t\in \left[t_{i1}^{0},t_{i-end}^{0}\right] \end{array}\right. \end{array}$$

where $\bar{V}$ is the average speed. *j* is the *j*th vehicle. *n* is the total number of vehicles.

The speed of the vehicle affects the optimization of phase offset and the calculation of the bidirectional cycle green wave bandwidth to a certain extent. During green wave coordinated control, variations in phase offset between intersections can result in a shift in the green wave bandwidth, which in turn affects the speed of the guided vehicle.

(b) Bidirectional cycle green wave bandwidth optimization

The control objective expression for the maximum number of vehicles passing in the green wave bandwidth time, as set out in Equations (23)–(25), was designed on the understanding that the intersection coordinated phase is of great importance for the release of traffic in different directions.

(23) $$P=\left|\sum_{m=1}^{N}\left[\lambda_{mz}\int_{b_{mzs}}^{b_{mze}}q_{mzd}\left(t\right)dt\int_{t_{i-end}^{0}}^{t_{i1}^{0}}a_{mz}\left(t\right)dt\right]-\sum_{m=1}^{N}\left[\lambda_{mf}\int_{b_{mfs}}^{b_{mfe}}q_{mfd}\left(t\right)dt\int_{t_{i-end}^{0}}^{t_{i1}^{0}}a_{mf}\left(t\right)dt\right]\right|$$

(24) $$H=\left\{\sum_{m=1}^{N}\left[\lambda_{mz}\int_{b_{mzs}}^{b_{mze}}q_{mzd}\left(t\right)dt\int_{t_{i-end}^{0}}^{t_{i1}^{0}}a_{mz}\left(t\right)dt\right]+\sum_{m=1}^{N}\left[\lambda_{mf}\int_{b_{mfs}}^{b_{mfe}}q_{mfd}\left(t\right)dt\int_{t_{i-end}^{0}}^{t_{i1}^{0}}a_{mf}\left(t\right)dt\right]\right\}$$

(25) $$\min B_{1}=\min\frac{P}{H}$$

where *P* represents the control objective expression of forward green wave bandwidth. *H* represents the control objective expression of reverse green wave bandwidth. *b_mzs_* denotes the start-time point of forward green wave bandwidth at the *m*th intersection. *b_mze_* denotes the end-time point of forward green wave bandwidth at the *m*th intersection. Where *b_mfs_* represents the start-time point of reverse green wave bandwidth at the *m*th intersection, *b_mfe_* represents the end-time point of reverse green wave bandwidth at the *m*th intersection. *q_mzd_* indicates the arrival rate of the forward traffic flow at the *m*th intersection. *q_mfd_* indicates the arrival rate of the reverse traffic flow at the *m*th intersection. *a_mz_* denotes the acceleration of the forward traffic flow. *a_mf_* denotes the acceleration of the reverse traffic flow. *λ_mz_* is the passing coefficient of the forward traffic flow at the *m*th intersection. *λ_mf_* is the passing coefficient of the reverse traffic flow at the *m*th intersection. *N* is the number of intersections.

The discretized control objective is calculated by Equations (26)–(28).

(26) $$p=\left|\sum_{m=1}^{N}\left[\lambda_{mz}\sum_{i=1}^{z}q_{mzd}^{b_{mzs}}(i)\sum_{i=1}^{z}v_{mz}^{t_{i1}^{0}}(i)\right]-\sum_{m=1}^{N}\left[\lambda_{mf}\sum_{i=1}^{f}q_{mfd}^{b_{mfs}}(i)\sum_{i=1}^{f}v_{mf}^{t_{i1}^{0}}(i)\right]\right|$$

(27) $$h=\left\{\sum_{m=1}^{N}\left[\lambda_{mz}\sum_{i=1}^{z}q_{mzd}^{b_{mzs}}(i)\sum_{i=1}^{z}v_{mz}^{t_{i1}^{0}}(i)\right]+\sum_{m=1}^{N}\left[\lambda_{mf}\sum_{i=1}^{f}q_{mfd}^{b_{mfs}}(i)\sum_{i=1}^{f}v_{mf}^{t_{i1}^{0}}(i)\right]\right\}$$

(28) $$\min B_{1}=\min\frac{p}{h}$$

where *z* is the number of time slots in the forward green wave bandwidth. *f* is the number of time slots in the reverse green wave bandwidth. $q_{mzd}^{b_{mzs}}(i)$ denotes the arrival rate of traffic at the *m*th intersection with green wave start time *b_mzs_* in the forward direction for the *i*th period. $q_{mfd}^{b_{mfs}}(i)$ denotes the arrival rate of traffic at the *m*th intersection with green wave start time *b_mfs_* in the reverse direction for the *i*th period.

The optimization goal is to balance bidirectional green wave bandwidths by minimizing the disparity in vehicle throughput between forward and reverse directions. Furthermore, the maximum number of vehicles passing through the overall green wave bandwidth is obtained by summing the number of vehicles passing through both the forward and reverse green wave bandwidths. However, the variation in demand for bidirectional traffic control is indicated by the inclusion of traffic passing coefficients, which demonstrate the varying levels of significance of bidirectional traffic flow. In practical applications, the traffic passing coefficients of each intersection can be set according to the actual traffic demand, as observed through empirical data. To illustrate, the disparate control requirements of outbound and inbound traffic on the corresponding arterials during the morning and evening peaks can be incorporated. This can be achieved by dividing the bidirectional traffic flow data into different time periods and designing the corresponding bidirectional traffic passing coefficients for each period. It is necessary that the coefficients satisfy the relationship shown in Equation (29).

(29) $$\lambda_{mz}+\lambda_{mf}=1$$

(c) Model establishment

The conventional approach to determining the green wave bandwidth is to calculate the duration of the green wave band based on the spatiotemporal trajectories of the first and last vehicles that pass through the coordinated control system without stopping along the coordinated control direction. However, the proposed method actively guides the vehicle within a specified range prior to reaching the stop line at the intersection. Therefore, the traditional method of obtaining the green wave bandwidth is not applicable to the present study. To optimize the bidirectional comprehensive green wave bandwidth, we use the green wave bandwidth optimization design software V1.0. Based on the multi-level speed guidance collaborative model, it is employed to optimize the phase offsets between the intersections.

In arterial green wave coordination control, the green wave bandwidth is typically modified in response to alterations in the phase offset. Consequently, the process of identifying the optimal green wave bandwidth for bidirectional cycle coordination requires the identification of the most effective combination of phase offsets between intersections. The variable of interest is the phase offset of each intersection, which ranges from 0 to *C*, where *C* represents the cycle time. The objective of the optimization process is to identify the maximum value for the green wave bandwidth, denoted by maxb. The DQNGA algorithm offers a significant advantage in the resolution of combinatorial optimization problems. The model considers the importance of traffic release in different phases of directional coordination at each associated intersection. Accordingly, the phase offsets between the associated intersections are calculated in order to obtain the optimal comprehensive green wave bandwidth for bidirectional cycles. The objective of controlling the maximum number of vehicles passing continuously within the comprehensive green wave bandwidth has been successfully achieved.

The control variables for this study are the vehicle guidance speed and the bidirectional cycle green wave coordinated phase offset. The objective of constructing the collaborative optimization model is to achieve the maximum average vehicle speed and the bidirectional cycle comprehensive green wave bandwidth. The objective function is presented in Equation (30).

(30) $$\begin{array}{l} \max Z=\psi \bar{V}+\zeta b\\ s.t.\;\left\{\begin{array}{l} V_{ij}^{end}=V_{\max}\\ t_{ij}^{end}=t_{i1}^{end}+\sum_{n=2}^{j}h_{in}\\ l_{ij}^{end}=l_{i1}^{end}\\ h_{ij}\geq \frac{S_{0}+len_{i\left(j-1\right)}}{V_{ij}^{end}}\geq h_{t}\\ v_{i}(t)\leq V_{\max},\;t\in \left[t_{i1}^{0},t_{i-end}^{0}\right]\;\\ v_{i}(t)\geq V_{\min},\;t\in \left[t_{i1}^{0},t_{i-end}^{0}\right]\\ \min\frac{p}{h}\leq b\leq \max b\\ g=\min\left[\max(g_{m-1,m},g_{m,m+1}),g_{\max}\right]\\ g_{m-1,m}^{\min}\leq g_{m-1,m}=\frac{Len_{m-1,m}^{opt}}{v_{m-1,m}^{pass}}+\frac{n_{m-1,m}^{pass}}{n}t_{m-1,m}\leq g_{m-1,m}^{\max}\\ g_{m,m+1}^{\min}\leq g_{m,m+1}=\frac{Len_{m,m+1}^{opt}}{v_{m,m+1}^{pass}}+\frac{n_{m,m+1}^{pass}}{n}t_{m,m+1}\leq g_{m,m+1}^{\max}\\ g^{\prime }=(C-g-y)\frac{q_{z\;or\;f}^{\max}}{\sum_{I=1}^{s}q_{z\;or\;f}^{\max}}\\ {g^{\prime }}_{\min}\leq g^{\prime }\leq {g^{\prime }}_{\max}\\ T_{j\_n}>0\\ t_{delay}^{\min}\leq t_{delay}\leq t_{delay}^{\max} \end{array}\right. \end{array}$$

where Z is the total objective function. ψ and ζ are the weights of average vehicle speed and comprehensive green wave bandwidth, respectively. If there is no queue at the stop line, the vehicles’ fleet will pass through the stop line at a speed defined as $v_{m-1,m}^{pass}$. *g_m_*_−1,*m*_ is the required green time from *m* − 1th intersection through *m*th intersection. *g_m_*_,*m*+1_ is the required green time from *m*th intersection through *m* + 1th intersection. $Len_{m-1,m}^{opt}$ is the length of the vehicles’ fleet from *m* − 1th intersection through *m*th intersection. $Len_{m,m+1}^{opt}$ is the length of the vehicles’ fleet from *m*th intersection through *m* + 1th intersection. *t_m_*_−1,*m*_ is the time when the vehicle enters the control area from *m* − 1th intersection through *m*th intersection. *t_m_*_+1,*m*_ is the time when the vehicle enters the control area from *m*th intersection through *m* + 1th intersection. $n_{m-1,m}^{pass}$ is the number of vehicles passing *m*th intersection continuously without stopping from the *m* − 1th intersection. $n_{m,m+1}^{pass}$ is the number of vehicles passing *m* + 1th intersection continuously without stopping from the *m*th intersection. *n* is the total number of vehicles. *g* is the green time of coordinated direction. *g*_max_ is the maximum green time. *g′* is the green time of uncoordinated direction. *C* is the cycle time. $q_{z\;or\;f}^{\max}$ is the maximum single-lane traffic flow of forward or reverse direction. *I* is the number of phases. *s* is the total number of phases. *y* is the total lost time, including amber light time and start losing time of all phases. *g_m_*_−1,*m*_ and *g_m_*_,*m*+1_ satisfy the constraints of the corresponding maximum and minimum green time. Meanwhile, *g′* satisfies the constraints of the corresponding maximum and minimum green time.

#### 3.2.3. Calculation Method of the Collaborative Model

**Figure 7:**
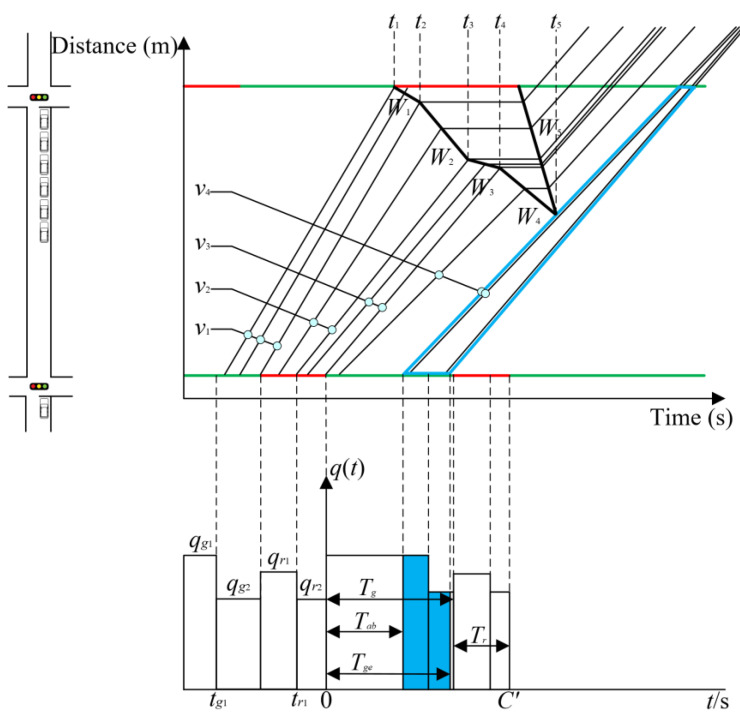
Vehicles travelling on the lane between intersections.

The objective of the present study is to ascertain the impact of phase offset, or red-light queue dissipation time at downstream intersections, on the green wave bandwidth. The number of vehicles passing in the green wave bandwidth with different phase offsets is calculated, and a suitable phase offset is selected with the objective of maximizing the number of vehicles passing in the comprehensive green wave bandwidth. In the context of green wave coordinated control, it is assumed that no secondary queuing occurs. The vehicles are assumed to travel between the upstream and downstream intersections in accordance with the illustration in Figure 7.

In order to achieve the objective of maximizing the number of vehicles passing through the green wave coordinated bandwidth, it is necessary to calculate *T_gb_* and *T_ge_* when the phase offset is determined. To facilitate the description of the calculation method, the definitions of the variables are presented in Table 2.

Step 1: The green time of each phase at the upstream intersection is divided according to the different traffic flows (e.g., *q_g_*_1_, *q_g_*_2_, *q_r_*_1_ and *q_r_*_2_ in Figure 7), and the traffic flows and the corresponding average speed of vehicles in each time period are determined (e.g., *v*_1_, *v*_2_, *v*_3_ and *v*_4_ in Figure 7).

Step 2: At the determined phase offset, the virtual travel lane is plotted to downstream intersection *i* + 1 by the average speed corresponding to each period during the green light time of the coordinated phase at upstream intersection *i*.

Step 3: Search for the driving line that encounters the red time at downstream intersection *i* + 1 for the first time in the above virtual driving line. Mark the starting time of the red light at the downstream intersection *i* + 1 corresponding to this driving line as *t*_1,*i*+1_, and the green time at the upstream intersection *i* corresponding to this driving line as *t_gbi_*.

Step 4: The driving lines corresponding to each time point are plotted separately for one cycle after starting from *t_gbi_* corresponding to the upstream intersection *i*. The driving lines at each time point are also plotted as they encounter the gather–disperse wave speed line at the downstream intersection *i* + 1 to determine *t_m_*_,*i*+1_.

Step 5: Determine whether the driving line passes through the interval enclosed by *t_m_*_,*i*+1_ during each period of the green time of upstream intersection *i*. If not passed, record the green time *T_gb_*_,*i*_ of the upstream intersection *i* corresponding to the first line. The green time *T_ge_*_,*i*_ of the last line corresponding to the upstream intersection *i*. Record the green time $T_{gb,i+1}^{c}$ of the first line corresponding to the downstream intersection *i* + 1, and the green time $T_{ge,i+1}^{c}$ of the last line corresponding to the downstream intersection *i* + 1.

Step 6: Obtain the final ${T^{\prime }}_{gbi}$ and ${T^{\prime }}_{gei}$ of the *N*th intersections corresponding to the public green wave band within the coordinated range: taking the *i* + 1th intersection as an example, if $T_{gb,i+1}^{c}>T_{gb,i+1}$, then $T_{gb,i+1}^{c}-T_{gb,i+1}=0$. If $T_{ge,i+1}^{c}<T_{ge,i+1}$, then $T_{ge,i+1}^{c}-T_{ge,i+1}=0$. On this basis, $\left[T_{gb,i+1},T_{ge,i+1}\right]$ is taken as the intersection *i* + 1, executes the effective green time of green wave coordinated control, and executes Step 5 again to obtain the updated $T_{gb,i+1}$ and $T_{ge,i+1}$. Meanwhile, obtain $T_{gb,i+1}^{c}$ and $T_{ge,i+1}^{c}$, and so on until road intersection *N*.

Step 7: Backtrack from *T_gb_*_,*N*_ and *T_ge_*_,*N*_ of intersection *N* and update *T_gb_*_,*i*+1_ and *T_ge_*_,*i*+1_ (*i* = *N* – 1, *N* − 2, ..., 1) in turn.

When applying the proposed method for bidirectional cycle green wave coordinated control, it is important to note that the variables of each intersection, including *q_g_*_1_, *q_g_*_2_, *q_r_*_1_ and *q_r_*_2_, *t_g_*_1_ and *t_r_*_1_, may change due to variations in control parameters. Therefore, it is necessary to update the coordinated phase offset of the green wave to ensure effective control. Thus, the phase offset optimization calculation needs to be redone based on the updated parameter values. To solve this issue, this paper proposes a cyclic optimization method for the phase offset at control cycle intervals. The steps are outlined below.

Step 7.1: The traffic flow in the control cycle obtained from the connected vehicle environment before the implementation of the green wave is divided, and the values of *q_g_*_1_, *q_g_*_2_, *q_r_*_1_, *q_r_*_2_, *t_g_*_1_, *t_r_*_1_ are recorded.

Step 7.2: Determine the optimized phase offset.

Step 7.3: Green wave coordinated control is implemented.

Step 7.4: The traffic flow in the control cycle obtained from the connected vehicle environment after the implementation of the green wave is divided, and the values of *q_g_*_1_, *q_g_*_2_, *q_r_*_1_, *q_r_*_2_, *t_g_*_1_, *t_r_*_1_ are recorded.

Step 7.5: Determine whether the difference of the above-mentioned variables compared to the previous one satisfies the requirements. If so, the method is finished. Otherwise, go to Step 7.2.

### 3.3. Bi-Level Combinatorial Optimization Method for Model Solving

**Figure 8:**
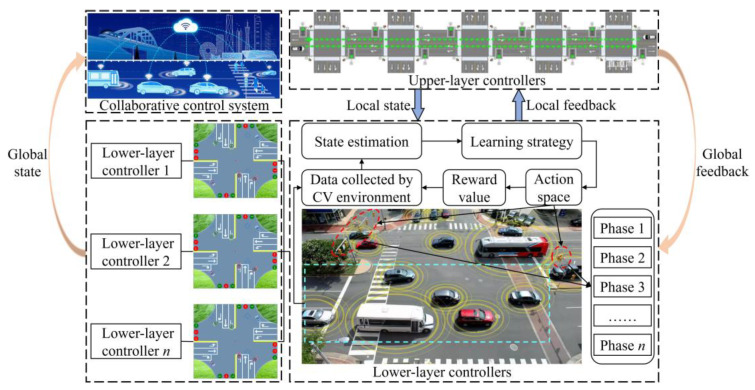
Bi-level combinatorial optimization method framework.

**Figure 9:**
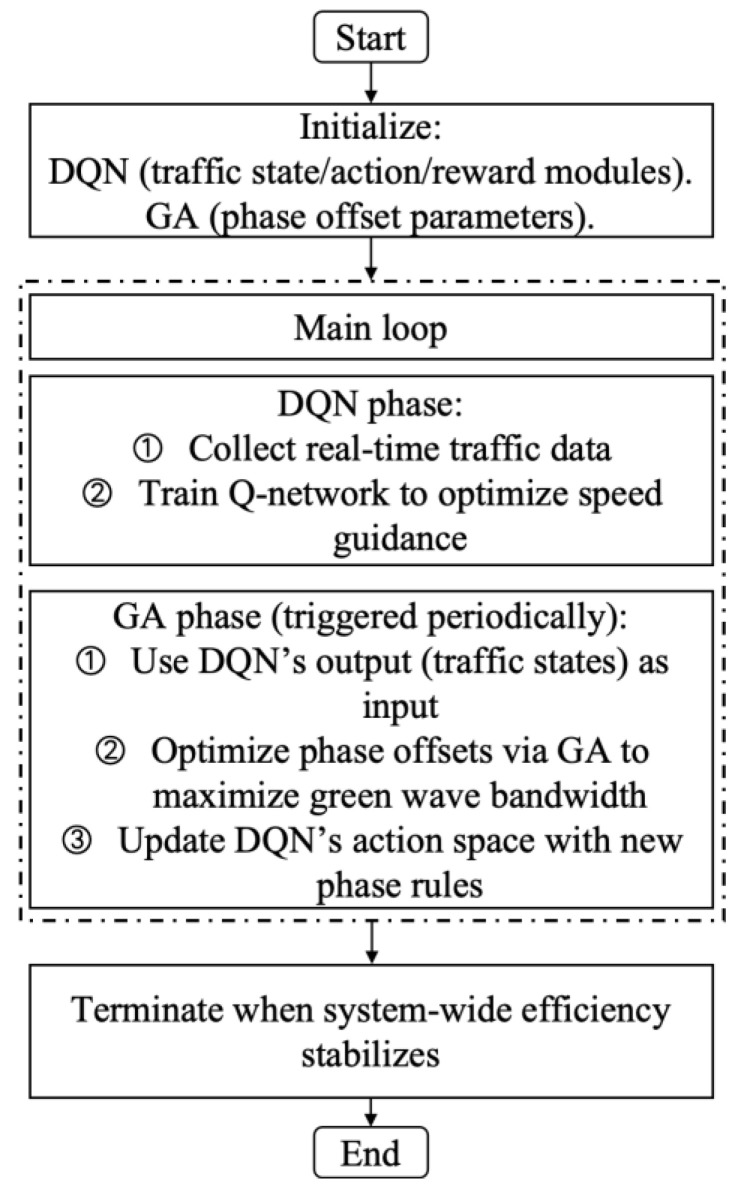
Flowchart of the DQNGA algorithm.

The bi-level combinatorial optimization method is implemented by gathering real-time signal timing data, vehicle travel information (such as speed and position), road traffic conditions, and other relevant data through the connected vehicle environment and traffic sensors. The data thus obtained are used to divide the collaborative combinatorial optimization control of the related intersection group into upper- and lower-layer control. The lower layer comprises multi-level vehicle speed guidance controllers at each intersection. Each controller employs a distinct learning strategy. The upper layer comprises bidirectional cycle green wave coordinated controllers, which adjust the temporary strategy of the lower layer primarily based on the feedback state of the lower layer. The upper- and lower-layer controllers jointly regulate the signals of the arterial roads within the study area. The bi-level combinatorial optimization method is illustrated in Figure 8. The following flowcharts have been designed to enhance the readability of the DQNGA algorithm described in this paper, which is shown in Figure 9.

#### 3.3.1. The Lower-Layer Control

**Figure 10:**
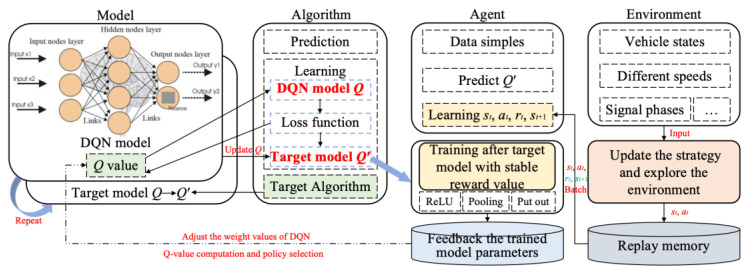
Framework of the lower-layer control based on DQN.

In contradistinction to static signal timing or heuristic approaches, the DQN-based framework is characterized by its capacity to adapt dynamically to fluctuations in traffic conditions, such as the influx of vehicles during phase transitions. It reduces the necessity for manual tuning through data-driven learning. It achieves hierarchical coordination via global–local rewards, prioritizing arterial road efficiency without disrupting access roads. The paper presents a methodology for selecting an action in the context of a connected vehicle environment, based on the state *s_t_* and reward *r_t_* transmitted by the connected vehicles during a specified time period *t*. The action set *A_t_* is used to select the appropriate speed to guide the vehicles at the intersection according to the current phase of the green time and phase transition state [49]. The state *s_t_* comprises the vehicle movement trajectories and their moving states in each segment of the related intersection groups. This information is transmitted by the connected vehicle environment. The reward, *r_t_*, is a form of feedback that is provided following the selection of an action, *a_t_*. The reward is based on several performance indexes, including the optimal guiding speed, the maximum average vehicle speed, and the maximum number of consecutive vehicles passing through the related intersection without stopping. Figure 10 shows the framework of the lower-layer control method. The DQN, which facilitates end-to-end learning, maps complex traffic states to optimal control actions, thereby overcoming the limitations of rule-based methods in dynamic environments. The discretization of control segments and multi-channel feature extraction have been shown to reduce dimensionality while preserving lane-specific behaviors. The integration of MDP with DRL ensures real-time adaptability while maintaining safety via phase transition rules. The global–local synergy effect is a further key element in preventing local optima, by penalizing actions that harm arterial throughput (for example, by reducing additional rewards when local decisions conflict with global goals).

(a) State space

In order to provide an accurate description of traffic conditions at arterial intersections, this study selects a number of parameters, including the operating state of vehicles in each direction of the intersection, their movement trajectories, and variations in signal phases, which are used as state inputs. Furthermore, the study area is divided into discrete units and modelled in order to accurately represent the specific distribution of position, speed and lane-change information for vehicles. The intersection groups are subdivided into discrete control segments, each of which is associated with a specific attention zone for connected vehicles. These segments are represented by a space characteristic matrix. The corresponding element in the space characteristic matrix is assigned a value of 1 when a vehicle is present in the free control segment of the lane, and a value of 0 otherwise.

The present study focuses on the distribution of vehicle positions and the associated lane-change information. A matrix is constructed to include the position of vehicles with multiple free control segments and the distance to the intersection. Moreover, the lane-change behavior of vehicles is also taken into account. This is determined based on the vehicles‘ movement trajectories and included in the vehicle lane-change characteristic matrix. In the event of a lane change, the element is recorded as 2; otherwise, it is recorded as 3.

In order to minimize computational effort, the speed of vehicles with multiple free control segments is stored in the speed characteristic matrix based on the vehicle operating state information detected by detectors. For each free control segment, the single-channel convolutional *Q* network detects the vehicle operating state information, including the position and speed of vehicles, during the time interval *t*. The corresponding elements are then recorded in the speed characteristic matrix. In the event that the aforementioned conditions are not met, the speed characteristic matrix is supplemented with a value of zero. Additionally, the current signal phase and its time variation are included in the state, as they are pivotal factors in the decision-making process. The aforementioned elements are then recorded in the aforementioned characteristic matrix.

(b) Action space

In order to enhance the efficacy of intersection groups, an intelligent control scheme of signals has been devised which establishes a flexible action space. The optimal speeds are selected to direct vehicular traffic at the intersection based on the prevailing phase of the green signal and the prospective phase transition state, taking into account all potential signal phases for each lane. The vehicle speed guidance strategy and feedback values are implemented based on a Markov Decision Process (MDP) mathematical model of the current phase of the green time and phase transition state. The MDP control strategy is integrated with iterative trials in deep reinforcement learning (DRL) to identify the optimal strategy for speed guidance when the feedback value is minimal. It should be noted that right-turn lanes are not included in the considerations, as they do not conflict with other lanes and are always accessible. Each loop represents a single phase of the green time and phase transition within a single phase cycle. The unit time of the loop is divided into discrete intervals. Subsequently, the currently active phase will be updated to the selected phase sequence state. The model establishes the maximum and minimum green times and their corresponding durations required to achieve phase transition. This signifies that a phase will transition to the subsequent phase upon the expiration of the maximum green light interval or in the event that the minimum green light interval is not satisfied. The original control scheme provides the basis for the iteration updates.

(c) Reward space

**Figure 11:**
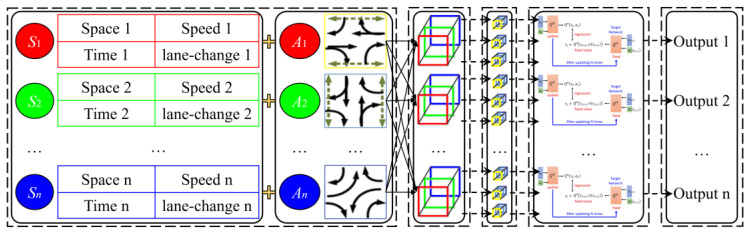
The hybrid learning framework of reward space.

In order to evaluate the efficacy of the selected action for each time interval and facilitate the vehicle’s adaptation to an optimal speed guidance strategy, the reward function furnishes global and local feedback on the performance of preceding actions. The local agent is responsible for monitoring and regulating the vehicle’s operations within a single intersection. Based on the observed vehicle operating states, the local agent generates and executes the necessary actions within the intersection, and subsequently outputs the current reward. In contrast, the global agent monitors the global state in order to assess the extent to which the local agent’s actions align with the overarching objective. Furthermore, additional rewards are provided to the local agent with the objective of enhancing the global efficiency of traffic flow at related intersection groups [44]. Figure 11 illustrates the hybrid learning framework of reward space.

The control objective of the local reward function is to maximize the average speed of vehicles, which allows for interactive and cooperative feedback optimization with the global agents [50]. The local reward (defined as $r_{t}^{local}$) includes the average speed reward and the additional reward, which is given in Equation (31).

(31) $$r_{t}^{local}=r_{t-\Delta t}^{avg\_speed}+r_{t}^{global\_add}$$

The $r_{t-\Delta t}^{avg\_speed}$ is calculated by Equation (32).

(32) $$r_{t-\Delta t}^{avg\_speed}=\max\left[\left(\sum_{j=1}^{n}\sum_{T=t-\Delta t}^{T_{n}}\frac{v_{jT}}{n}\right)-\left(\sum_{j=1}^{n}\sum_{T=(t-1)-\Delta t}^{T_{n-1}}\frac{v_{j(T-1)}}{n}\right)\right]$$

where $r_{t-\Delta t}^{avg\_speed}$ is the average speed reward. $r_{t}^{global\_add}$ is the additional reward. *v_jT_* is the speed of the *j*th vehicle within the time interval *T*. *v_j_*_(*T*−1)_ is the speed of the *j*th vehicle within the time interval *T* − 1. *j* is the *j*th vehicle. *n* is the total number of vehicles. Since the right-turn vehicles are not affected by the traffic light, the average speed reward will not include the right-turn vehicles. The average speed reward function is expressed as all vehicles except the right-turn vehicles at the intersection within the sliding time window Δ*t*. It is worth noting that the number of vehicles may still increase during the phase transition. So, Δ*t* should contain the amber light time.

When the actions of local agents impact the optimal speed at the global level, global agents reduce additional rewards and prevent local agents from enhancing the efficiency of the intersection, thereby reducing global rewards. This approach ensures an overall optimal speed guidance strategy in arterial coordination.

In order to guarantee objectivity, it is essential that the global reward is based exclusively on the state space of each local agent. The number of vehicles carried by the global agent should be adjusted for all lanes with the objective of maximizing continuous, non-stop throughput of the main arterial roads while avoiding any impact on traffic flow on the access roads. Meanwhile, in order to achieve the objective of maximizing the continuous non-stop throughput of the main arterial roads while avoiding any adverse impact on the traffic flow on the access roads, the global reward function (defined as $r_{t}^{global}$) is expressed as the continuous non-stop throughput [51]. The calculation is presented in Equation (33).

(33) $$\begin{array}{l} r_{t}^{global}=\max\left(\frac{N_{mainRd}^{T}}{M}-\frac{N_{mainRd}^{T-1}}{M}\right)\\ s.t.\;\left\{\begin{array}{l} N_{mainRd}^{T}=\sum_{T=t-\Delta t}^{T_{n}}N_{pass}^{T}+\left[\sum_{T=t-\Delta t}^{T_{n}}\left(N_{out\_accessRd}^{T}-N_{in\_accessRd}^{T}\right)\right]\\ N_{mainRd}^{T-1}=\sum_{T=(t-1)-\Delta t}^{T_{n-1}}N_{pass}^{T-1}+\left[\sum_{T=(t-1)-\Delta t}^{T_{n-1}}\left(N_{out\_accessRd}^{T-1}-N_{in\_accessRd}^{T-1}\right)\right] \end{array}\right. \end{array}$$

where $N_{mainRd}^{T}$ is the total number of vehicles passing through the main arterial road during the time period from *T* to *T* + 1. $N_{pass}^{T}$ is the number of vehicles continuously passing without stopping on the main arterial road during the time period from *T* to *T* + 1. $N_{out\_accessRd}^{T}$ is the number of vehicles leaving the access road and entering the main arterial road from time *T* to time *T* + 1. $N_{in\_accessRd}^{T}$ is the number of new vehicles entering the access road from time *T* to time *T* + 1. $N_{mainRd}^{T-1}$ is the total number of vehicles passing through the main arterial road during the time period from *T* − 1 to *T*. $N_{pass}^{T-1}$ is the number of vehicles continuously passing without stopping on the main arterial road during the time period from time *T* − 1 to time *T*. $N_{out\_accessRd}^{T-1}$ is the number of vehicles leaving the access road and entering the main arterial road from time *T* − 1 to time *T*. $N_{in\_accessRd}^{T-1}$ is the number of new vehicles entering the access road from time *T* − 1 to time *T*. *M* is the number of intersections.

Given the varying levels of importance attributed to access roads in comparison to the primary arterial routes, it is essential to assign distinct weights to the constituent elements of each. Equation (34) calculates the global reward function of main arterial roads and intersecting access roads at time interval *T* (defined as $R_{T}^{global}$), assuming that it contains ax elements.

(34) $$R_{T}^{global}=\sum_{\delta =0}^{1}\sum_{x=1}^{a_{x}}\chi_{\delta ,a_{x}}r_{\delta ,a_{x},t}^{global}$$

where *δ* is a symbol, and its value of 0 indicates an intersecting access road, otherwise, its value of 1 indicates a main arterial road. $\chi_{\delta ,a_{x}}$ is the weight coefficient. $r_{\delta ,a_{x},t}^{global}$ is the global reward value of element *a_x_*.

Equation (34) shows that to enable the continuous flow of vehicles through the intersection and ensure easy convergence of the model, $R_{T}^{global}$ must be maximized and $R_{T}^{global}>0$ during the learning process.

DQN algorithm

The fundamental component of the DQN model is a convolutional neural network. Training is conducted using Q-learning to obtain the output, which represents the estimated Q-value of the optimal speed guidance strategy. The Q-value represents the total rewards that an agent can obtain when acting in state *s_t_*. It can be approximated by selecting the action *A_t_*_+1_ that yields the maximum Q-value Q′, which is calculated using Equation (35).

(35) $$\begin{array}{ll} Q\left(s_{t},A_{t}\right) & =r_{t+1}+\mu r_{t+2}+\cdots +\mu^{m-1}r_{t+m}\\ & \approx r_{t+1}+\mu \underset{A}{\max}\left[Q^{\prime }(s_{t+1},A_{t+1}\left|\theta \right.),\theta^{\prime }\right] \end{array}$$

where $Q^{\prime }(s_{t+1},A_{t+1}\left|\theta \right.)$ is the *Q* value when an action *A_t_*_+1_ is selected under state *s_t_*_+1_ and the current network parameter *θ* is updated for replication to the target network *θ′*, *μ* is the discount rate that increases a penalization for future rewards compared to the immediate reward *r_t_*_+1_. The larger *μ* is, the more agents would focus on subsequent rewards, while, if *μ* is smaller, agents would pay more attention to the current reward. In order to provide stable updates in each iteration, a separate target network *θ′* is used to generate *Q* values. The parameters in the main neural network are updated by back propagation, where *θ′*. is updated based on *θ* in Equation (36).

(36) $$\theta^{\prime \prime }=\xi \theta^{\prime }+\left(1-\xi \right)\theta$$

where *ξ* is update rate, which indicates the impact of the new parameters on the target network degree.

Figure 10 illustrates the utilization of two neural networks by the DQN to enhance the stability of the training process. The *Q′* value represents the predicted value of the neural network (NN) based on a given input sample. In the DQN framework, two NNs will be employed to predict the Q values, with one derived from the base NN model and the other from the target NN model. The mean square error (MSE) between the two *Q′* values is employed as a loss function, facilitating the updating of the weights of the NNs. Following the completion of one episode, the weights of the base NN are copied or updated to the target NN with the objective of minimizing the loss function. This process reduces the error term of the network to a finite interval and ensures that the Q values are within a reasonable range. Consequently, all vehicles will attain a globally optimal speed at the pertinent intersection groups.

The loss function (defined as *J*(*θ*)) is expressed as the MSE between *Q′* predicted from the base NN and the target NN network, which is calculated by Equation (37).

(37) $$J(\theta )={\sum_{E}\frac{1}{N_{training}}\left\{\left[r+\mu \underset{A^{\prime }}{\max}\left[{Q^{\prime }}_{t}(s^{\prime },A^{\prime }\left|\theta \right.),\theta^{\prime }\right]-Q_{t}(s^{\prime },A^{\prime }\left|\theta \right.)\right]\right\}}^{2}$$

where *N_training_* is the number of times when the network is training.

In order to minimize the loss function *J*(*θ*), the Adaptive Moment Estimation (Adam) (i.e., a stochastic gradient descent method) is implemented. First, the ranking-based prioritized experience replay structure method is used to improve the learning efficiency. The calculation of the prioritized probability of samples involves increasing the replay probability of samples with an average vehicle speed in a ranking-based approach. The error (defined as $\beta_{v_{j}}$) of sample *v_j_* is calculated by Equation (38). $\beta_{v_{j}}$ arranges in order, and let the priority $p_{v_{j}}$ be the reciprocal of their order.

(38) $$\beta_{v_{j}}=\frac{p_{v_{j}}^{P_{\tau }}}{\sum_{j}p_{v_{j}^{all}}^{P}}\left|{\left(Q^{\prime }(s^{\prime },A^{\prime }\left|\theta \right.),\theta^{\prime }\right)}_{v_{j}}-Q{\left(s^{\prime },A^{\prime }\left|\theta \right.\right)}_{v_{j}}\right|$$

where $P_{\tau }$ is the number of using priority, when $P_{\tau }$ is 0, the random sampling is taken. $p_{v_{j}}^{P_{\tau }}$ is the priority of vehicle speed. $\sum_{j}p_{v_{j}^{all}}^{P}$ is the total number of vehicle speeds’ priority.

Second, the weights in the neural network are updated by the gradient of loss function with learning rate (defined as $\omega_{\beta_{v_{j}}}$), which is calculated by Equations (39)–(41). In addition, the ranking-based prioritized experience replay structure method is used to explore possible actions at the beginning of the training stages. The agent will randomly choose an action with probability of $y(p)=\frac{p_{v_{j}}^{P_{\tau }}}{\sum_{j}p_{v_{j}^{all}}^{P}}$. Otherwise, the agent will choose an action *A_t_*_+1_ that obtains the maximum *Q′* value predicted by the training neural network.

(39) $$\frac{\partial J(\theta )}{\partial \theta }=\sum_{E}\frac{1}{N_{training}}\left\{\left[r+\mu \underset{A^{\prime }}{\max}\left[Q(s^{\prime },A^{\prime }\left|\theta \right.),\theta^{\prime }\right]-Q(s,A\left|\theta \right.)\right]\frac{\partial Q(s,A\left|\theta \right.)}{\partial \theta }\right\}$$

(40) $$\theta_{t+1}=\theta_{t}-\omega_{\beta_{v_{j}}}\frac{\partial J_{t}}{\partial \theta_{t}}$$

(41) $$\omega_{\beta_{v_{j}}}=1-\omega_{\beta_{0}}\cdot \frac{h}{H}\cdot \frac{\frac{{\dot{p}}_{v_{j}}^{P_{\tau }}}{1-\rho_{v_{j}}^{P_{\tau }}}}{\sqrt{\frac{{\dot{p}}_{v_{j}^{all}}^{P}}{1-\rho_{v_{j}^{all}}^{P}}+\varpi }}$$

where $\omega_{\beta_{0}}$ is the initial learning rate. *h* is the current episode number. *H* is the total number of episodes. ${\dot{p}}_{v_{j}}^{P_{\tau }}$ is the updated first moment of $p_{v_{j}}^{P_{\tau }}$. ${\dot{p}}_{v_{j}^{all}}^{P}$ is the updated second moment of $p_{v_{j}^{all}}^{P}$. $\rho_{v_{j}}^{P_{\tau }}$ is the first exponential decay rate of $p_{v_{j}}^{P_{\tau }}$. $\rho_{v_{j}^{all}}^{P}$ is the second exponential decay rate of $p_{v_{j}^{all}}^{P}$. ϖ is the coefficient that gives stability to the value.

The detailed algorithms of the DQN are given in Algorithm 1.
**Algorithm 1** The pseudo code of DQN algorithmInitialize replay memory *D* with capacity *N*Initialize reward function *Q* with random weights *θ*Initialize target reward function *Q*′ with weights *θ′***For** episode = 1, *H* **do**Initialize current state *s_t_***For** *t* = 1, *T* **do**With learning rate $\omega_{\beta_{v_{j}}}$ select a random action *a_t_*Otherwise select $a_{t}=\arg\underset{a}{\max}Q(s_{t},a\left|\theta \right.)$   $\mathrm{Execute}\;\mathrm{action}\;a_{t}\;\mathrm{in}\;\mathrm{emulator}\;\mathrm{and}\;\mathrm{observe}\;\mathrm{reward}\;r_{t}=\{r_{t}^{local},r_{t}^{global}\}$
  Set next state *s_t_*_+1_  Store (*s_t_*, *a_t_*, $r_{t}^{K}$, *s_t_*_+1_) in *D*  Sample random minibatch of (*s_t_*, *a_t_*, $r_{t}^{K}$, *s_t_*_+1_) from *D*  **For** every (*s_t_*, *a_t_*, $r_{t}^{K}$, *s_t_*_+1_), *N* **do**  Set $Q_{t}=\left\{\begin{array}{ll} r_{t}, & \mathrm{If}\;\mathrm{episode}\;\mathrm{terminates}\;\mathrm{at}\;\mathrm{time}\;\mathrm{step}\;t\\ r_{t+1}+\mu \underset{A}{\max}\left[Q^{\prime }(s_{t+1},A_{t+1}\left|\theta \right.),\theta^{\prime }\right], & \mathrm{Otherwise} \end{array}\right.$   Calculate the loss function value *J*(*θ*)    Perform the Adaptive Moment Estimation   (including (38) ~ (40)) with minimizing *J*(*θ*) to update *θ*    Every *t* steps, copy weights from base NN to the target NN,     reset *Q*′ = *Q*  **End For** **End For****End For****Until** training completed

#### 3.3.2. The Upper-Layer Control

In terms of lower-layer control, the upper layer coordinates the control of related intersection groups based on predetermined conditions, such as cycle time, green ratio, and phase offset. Subsequently, the control scheme is then updated based on the initial and target states of the vehicles, as output by the lower layer. Upon adjustment of the phase offset, the aforementioned process is repeated, and the resulting green wave bandwidth after the adjustment is obtained. Consequently, the automatic adjustment of the phase offset to achieve the optimal bidirectional cycle-coordinated green wave bandwidth is essentially a problem of combining and optimizing the phase offset between intersections. The objective is to combine the phase offset of each intersection, which should take values in the range of [0, *C*]. The objective of the optimization process is min*B*_1_. The core process of combinatorial optimization is the search for the optimal green wave bandwidth.

Given the significant advantages of the genetic algorithm (GA) in solving combinatorial optimization problems, it is decided to use the GA to obtain the phase offsets between intersections. This is done to achieve the control objective of considering the importance of traffic release in different directions with coordinated phases at intersections and the maximum number of consecutive passing vehicles within the green wave bandwidth.

The GA is designed to determine the optimal coding strategy for the phase offset variable, which has a value range of [0, *C*]. However, the value of *C* may differ in various traffic environments and may not necessarily satisfy the 2^n^ condition. When a variable contains finite (non-2^n^) discrete effective values, some common binary codes may exhibit redundancy. To enhance the algorithm’s complexity, special mechanisms such as fixed remapping, random remapping, and probabilistic remapping should be employed to map redundant codes into effective codes. Consequently, the real number coding method is an effective means of resolving combinatorial optimization problems.

The procedure of the real number coding genetic algorithm is given in Algorithm 2.
**Algorithm 2** The procedure for GA with real number coding**Begin** **Initialization:** {  Select the type of genetic operation and determine the parameters such as  crossover probability and mutation probability.  Set evolutionary algebra counter *t* = 0.  The initial population *B*(0) = {*O*_1_, *O*_2_, …, *O*_N_} is   generated by constructing chromosomes with   phase offsets between associated intersections as   genes, where *N* represents the population size. }  Measurement: select min*B*_1_ as the objective function, and then the fitness value  of each individual is calculated by the initial population *B*(0).  **While** (the termination conditions are not satisfied) **do**  {    **Crossover:** *B*(*t*) is performed crossover operation to generate population    *B′*(*t* + 1).    **Mutation:** *B′*(*t* + 1) is performed mutation operation to generate population    *B″*(*t* + 1).    **Measurement:** The fitness value of each individual in population *B″*(*t* + 1) is     calculated.    **Selection:** *A*∪*B″*(*t* + 1) is selected to generate a new population *B*(*t* + 1), where    *A* represents a subset or empty set of *B*(*t*).    *t* = *t* + 1.  }**End**

## 4. Case Study

### 4.1. Simulation Scenario

In order to eliminate the effects of road section peculiarities and the periodic signal timing plan, two road sections are randomly selected from the arterial coordinated road sections in Yangzhou. The result is validated by examining the arterial coordinated control of the aforementioned road sections over multiple time periods throughout the day. Traffic data were collected from microwave radar sensors and onboard diagnostics (OBD) devices deployed by Yangzhou Traffic Management Bureau. The scenario in the case study has a total of 68,342 connected vehicles. Vehicle trajectories, speeds, and signal states were synchronized via the VISSIM COM interface to ensure spatiotemporal consistency. The data pertaining to the connected vehicles are presented in Table 3. Data collection and sensor traffic data are collected via the following methods:

**On-board GPS:** 68,342 connected vehicles with 1 Hz sampling and GPS timestamps.

**Roadside Cameras:** These cameras were utilized to capture lane-specific vehicle counts at intersections, validated against roadside LiDAR measurements (±2% error).

**VISSIM COM Interface:** Simulated vehicle trajectories with 0.1 s resolution.

The data covered three periods (morning/evening/evening peaks) on arterial roads in Yangzhou, China.

**Figure 12:**
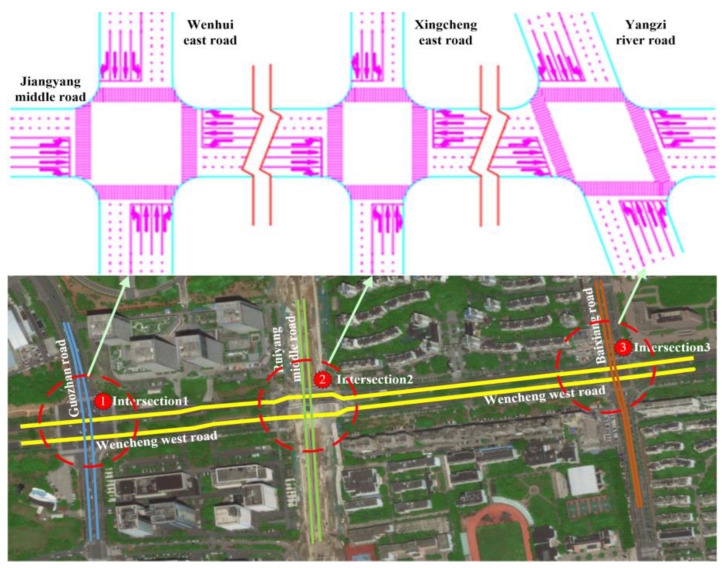
VISSIM visual interface of Jiangyang middle road section.

**Figure 13:**
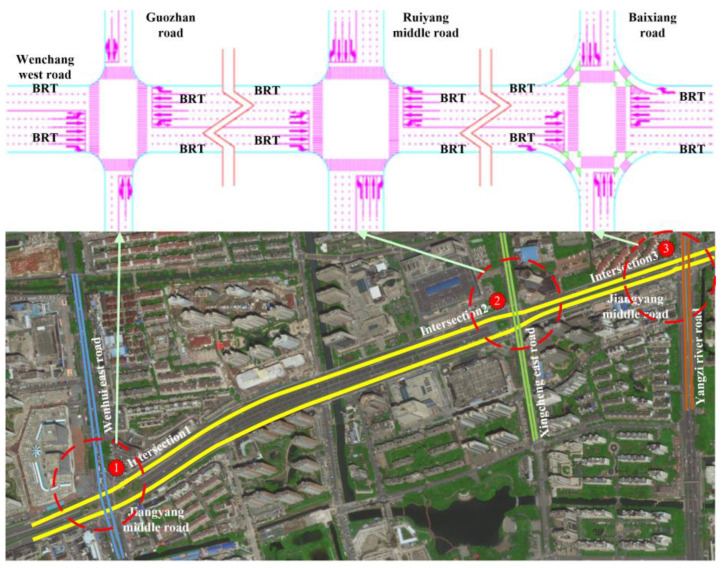
The VISSIM visual interface of Wenchang road section.

As illustrated in Figure 12 and Figure 13, the VISSIM simulation platform is constructed upon a three-dimensional Google map. Figure 12 shows the region containing all sections within Jiangyang middle road. The yellow line is the Jiangyang middle road, while the blue line represents the Wenhui east road. The green line is Xingcheng east road. The orange line represents the Yangzi river road.

Figure 13 illustrates the spatial distribution of the various sections within Wenchang west road, The yellow section represents Wenchang west road, while the blue section represents Guozhan road. The green section represents Ruiyang middle road, while the orange section represents Baixiang road.

**Figure 14:**
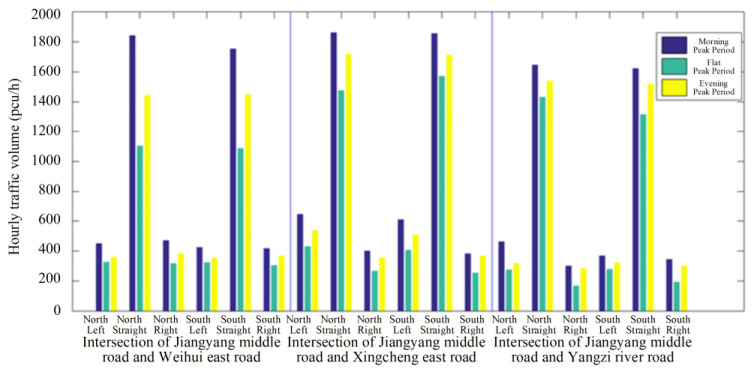
The traffic volume of Jiangyang middle road section’s intersections in each period.

**Figure 15:**
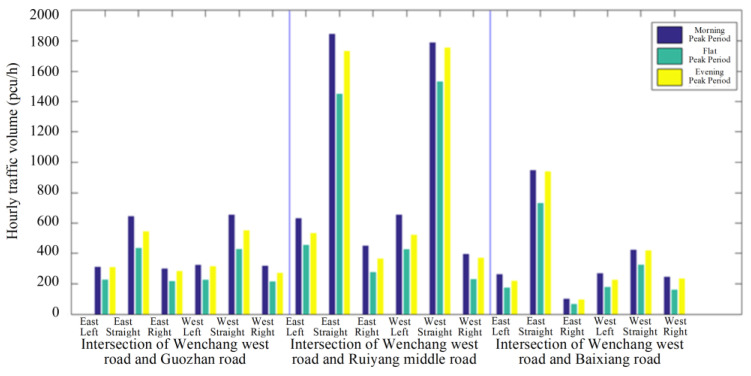
The traffic volume of Wenchang west road section’s intersections in each period.

When three periods (morning peak, flat peak and evening peak) are observed, the traffic volume with the arterial coordinated direction of two related road sections is as shown in Figure 14 and Figure 15.

Figure 14 shows that the traffic volume in the north–south straight direction on the Jiangyang middle road section is large. Figure 15 depicts the substantial traffic volume in the east–west direction on the Wenchang west road section. With regard to the relevant intersection groups on the Jiangyang middle road, the north-south straight phase has been designated as the arterial coordination phase. The north–south direction is the primary arterial road with forward traffic flow, while the south–north direction is the primary arterial road with reverse traffic flow. For the related intersection groups of Wenchang west road, the east–west straight phase is designated as the arterial coordination phase. The east–west direction is the primary arterial road with forward traffic flow, while the west–east direction is the primary arterial road with reverse traffic flow.

### 4.2. Simulation Verification

In order to guarantee the reliability of the proposed model, a bidirectional cycle green wave coordinated control and multi-level vehicle speed guidance platform is constructed using VISSIM 4.30/MATLAB 2025a simulation fused with real traffic data. The MAXBAND model is employed as a reference for the simulation, while the optimization function is obtained through the DQNGA algorithm, and the results are obtained through VISSIM via the COM interface. Furthermore, the COM interface can be adjusted to simulate the connected vehicle environment and achieve multi-level speed guidance through secondary development. The practical constraints are determined based on the experimental scenario. It is initially assumed that the normal speed of vehicles during the peak period is 20 km/h, and that during the flat peak period, the normal speed is 40 km/h. The speed limit of the road section is selected as the maximum speed. The starting point of the speed adjustment area is set at 300 m before the intersection. A speed guidance simulation is conducted for the two related road sections during the morning peak, flat peak and evening peak periods under MAXBAND and the proposed model. The average speed between each intersection of each related road section is calculated, and then the phase offset between each intersection is optimized using the optimization software to obtain the corresponding bidirectional cycle comprehensive green wave bandwidth. The newly devised green wave coordination plan is input into the vehicle speed guidance simulation platform and simulated repeatedly until the average vehicle speed and the bidirectional cycle comprehensive green wave bandwidth have reached their optimal values.

#### 4.2.1. Results of DQN with Speed Guidance

A primary convolutional neural network is selected to provide state space feedback values, thereby enabling the selection of the most valuable action. Initially, DQN generates a single training batch of data and stores the current state and action. The feedback values are stored as quaternions (*s*, *a*, *r*, *s′*) in the replay memory D. The target neural network (NN) is a separate neural network that increases the learning stability and obtains the optimal speed guidance strategy by selecting the action with the maximum *Q* value and updating the priority of the samples after each training. Then, the learning rate in the NN is updated by Adam back propagation. The model derives the initial control scheme based on the initial learning rate and the action with the maximum *Q* value feedback operation. Finally, the bidirectional cycle comprehensive green wave bandwidth at all related intersection groups is adjusted according to the optimal vehicle speed. Thus, the Rectified Linear Unit (ReLU) activation function is used for all hidden layers, while the linear activation function is applied to the output layer. The Adam optimization algorithm [52] is employed to train the NN models. The parameters of the DQN model for the purposes of model training are presented in Table 4.

**Figure 16:**
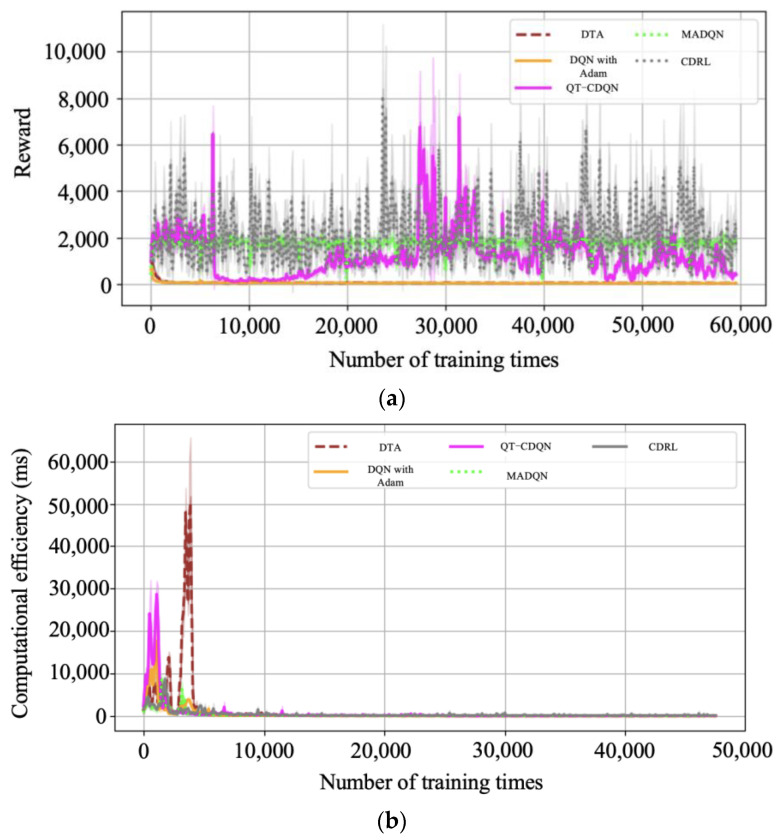
Performance results with different algorithms. (**a**) The results of reward with different algorithms. (**b**) The results of computational efficiency with different algorithms.

In order to test the performance of the proposed method, as illustrated in Figure 16, the outcomes are compared with those of alternative algorithms, including Coordinated Deep Reinforcement Learners (CDRL) [52], Multi-agent Deep Q-learning (MADQN) [53], Cooperative Deep Q-network with Q-value Transfer (QT-CDQN) [54] and Dual Targeting Algorithm (DTA) [55].

As demonstrated in Figure 16, the Adam-optimized DQN achieves superior performance compared to baseline algorithms, as evidenced by lower delays and higher throughput, indicating that the agents in the proposed algorithm employ a more effective speed guidance strategy than the other algorithms, resulting in optimal outcomes. In order to ensure convergence stability, an integrated modified reward function based on the local reward function and global reward function is employed. The function attempts to maximize the reward obtained in each iteration in isolation. However, CDRL is based on the same structure that has been trained using transfer learning. The MADQN algorithm is characterized by an agent that is trained independently by the deep Q-learning algorithm, with no cooperation among these agents. The QT-CDQN algorithm demonstrates superior learning and convergence rates compared to both MADQN and CDRL. In comparison to both CDRL and MADQN, as well as QT-CDQN, DTA is capable of achieving faster convergence and superior stability.

**Figure 17:**
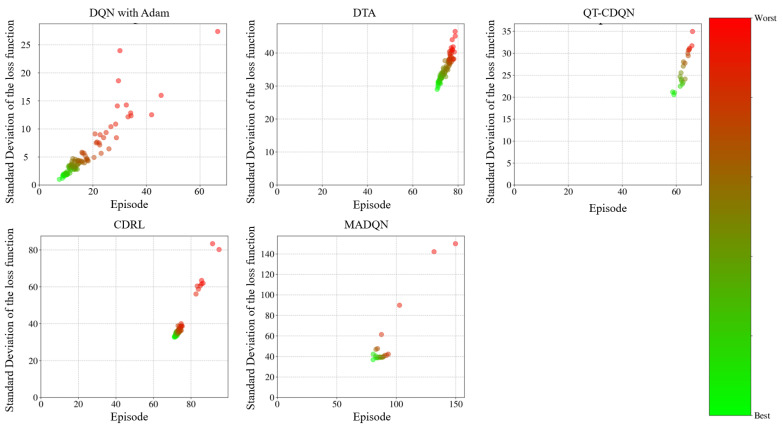
The standard deviation results of loss function under different discount rates.

**Figure 18:**
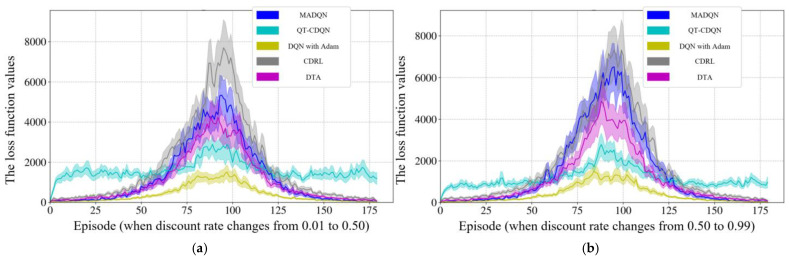
The loss function curves under different discount rates at the Jiangyang middle road section. (**a**) The loss function curves when discount rate changes from 0.01 to 0.50 at the Jiangyang middle road section. (**b**) The loss function curves when discount rate changes from 0.50 to 0.99 at the Jiangyang middle road section.

**Figure 19:**
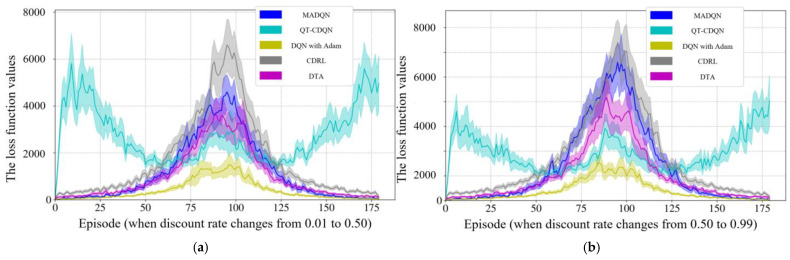
The loss function curves under different discount rates at the Wenchang road section. (**a**) The loss function curves when discount rate changes from 0.01 to 0.50 at the Wenchang road section. (**b**) The loss function curves when discount rate changes from 0.50 to 0.99 at the Wenchang road section.

The Adam is introduced in DQN with the objective of achieving a balance between exploration and exploitation of actions and states. It is generally expected that the DQN training procedure will explore a greater number of potential actions based on states at the outset, with exploitation becoming more prevalent as action strategies are refined. The DQN with Adam learning procedure is capable of obtaining previous action strategies from previous scenarios, which enables the training procedure to converge on optimal values by exploring all possible actions. To test this hypothesis, the loss function curves are examined under different discount rates at the Jiangyang middle and Wenchang road sections. The ensuing results of the training are presented in Figure 17, Figure 18 and Figure 19. As demonstrated in Figure 16, the selection of hyperparameters exerts a substantial influence on the performance of a designated traffic signal control. The general trend observed in Figure 16 is that methods with a higher number of hyperparameters (e.g., DTA, QT-CDQN, CDRL, MADQN) demonstrate a greater difference in performance than methods with a lower number of hyperparameters (e.g., DQN with Adam).

It can be observed that the DQN with Adam is capable of achieving a comparable level of stability in reward with full exploration (discount rate changes from 0.01 to 0.99), which suggests that the proposed method is efficacious in the Jiangyang middle road section and the Wenchang road section. Figure 18 and Figure 19 illustrate the loss function values of queues and delays for each intersection. These results are consistent with those previously observed with the hyperparameter search and travel data. However, readers should note the comparison of the performance of the DQN with Adam and DTA, QT-CDQN, CDRL, and MADQN in Figure 18 and Figure 19. The DQN with Adam achieves relatively low queues and latencies at the beginning and end of the simulation, whereas it outperforms the other four algorithms when the demand peaks in the middle of the simulation. In scenarios characterized by high traffic demand, the DQN with Adam has the capacity to select the subsequent phase in a non-periodic manner. This ability may facilitate a reduction in queues and delays that exceeds the capacity of the four algorithms, which are constrained by a periodicity. However, it is noteworthy that the performance of the four algorithms is compromised during periods of low demand, a time when formulating an optimal policy should be a relatively straightforward task. It can be hypothesized that the other four algorithms may have overfitted to periods of the environment where the magnitude of the reward is significant (i.e., the middle of the simulation, when the demand is at its peak) and converged to a policy that is not well-suited to the environment during times of low traffic demand.

**Figure 20:**
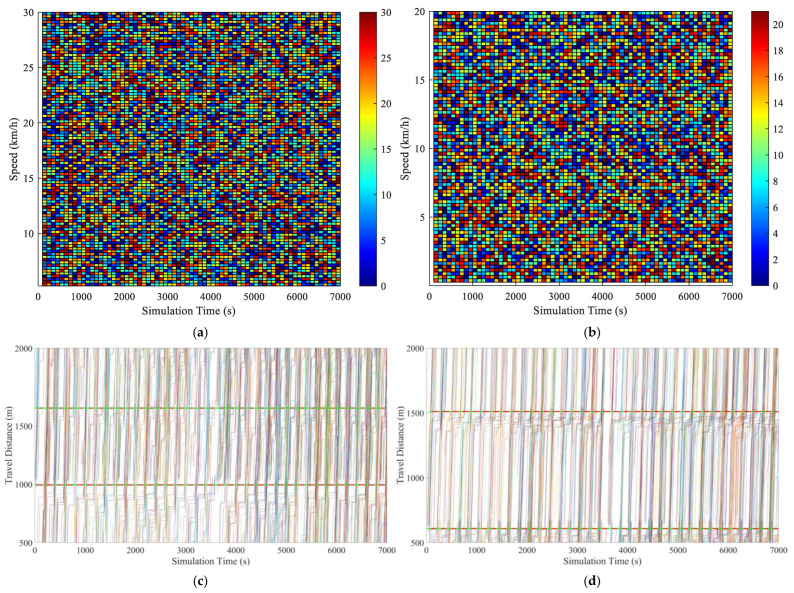
The trajectories of the MAXBAND model with Jiangyang middle and Wenchang road section. (**a**) The trajectories of Jiangyang middle road section. (**b**) The trajectories of Wenchang road section. (**c**) The trajectories of Jiangyang middle road section; (**d**) The trajectories of Wenchang road section.

**Figure 21:**
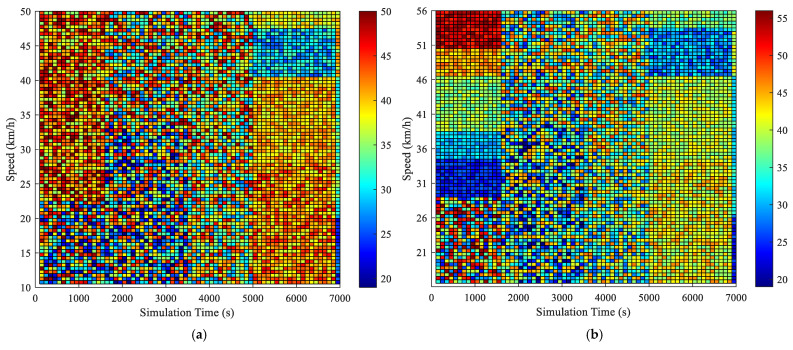

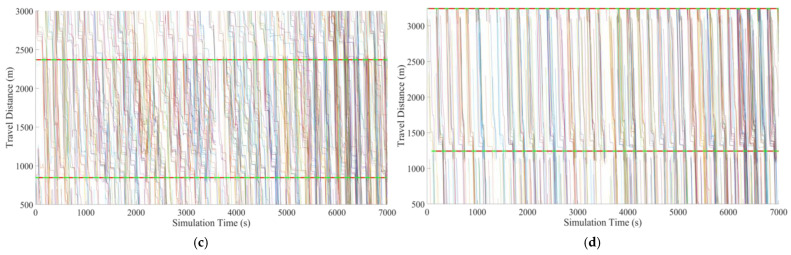
The speeds and trajectories of the proposed model with Jiangyang middle and Wenchang road section. (**a**) The speeds of Jiangyang middle road section. (**b**) The speeds of Wenchang road section. (**c**) The trajectories of Jiangyang middle road section. (**d**) The trajectories of Wenchang road section.

Figure 20 and Figure 21 illustrate the trajectory and speed results of MAXBAND and the proposed models. The time axis indicates the phase of intersection 1 transferring to green, followed by vehicles entering the current road section. Additionally, the heat values in the figure indicate the speed magnitude at the current time. As shown in Figure 20a, the MAXBAND model causes vehicles to stop at intersection 1 of the Jiangyang middle road section, resulting in the formation of a queue at approximately 18 s. The queue begins to dissipate at 46 s. Meanwhile, Figure 20b demonstrates that vehicles halt at the intersection of the Wenchang Road section, resulting in the formation of a queue at approximately 12 s. Upon the transition to the subsequent phase, the queue begins to dissipate at 27 s. As demonstrated in Figure 20c,d, vehicles are observed to wait at the stop line and queue for each cycle in the MAXBAND model, resulting in significant traffic delays at the related intersection groups.

As demonstrated in Figure 21a,b, the proposed model indicates that connected vehicles have the capacity to travel collectively as a fleet. The speed of the vehicle fleet is optimized based on the current traffic signal timing, resulting in a speed of approximately 40–45 km/h, which is higher than the speed of traffic free flow. This process leads to the formation of minimal queues, thereby enabling the majority of vehicles to pass through the relevant intersection groups without halting. In contrast, Figure 21c,d demonstrate that the majority of vehicles traverse the associated intersection groups without stopping. Conversely, the majority of the curves remain parallel throughout the entire process, with minimal delays observed under the proposed model.

#### 4.2.2. The Results of GA with the Bidirectional Cycle Comprehensive Green Wave Bandwidth

**Figure 22:**
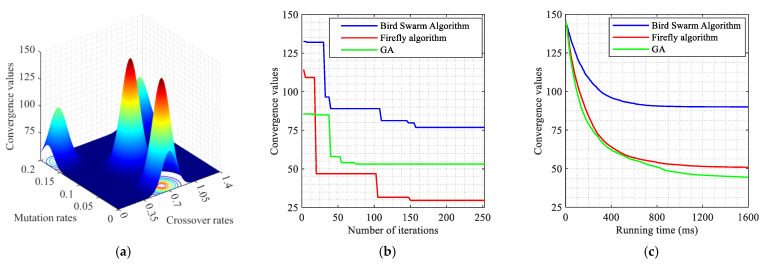
The convergence curves, fitness function variations and computational time of GA with different algorithms. (**a**) The fitness function variations. (**b**) The convergence curves. (**c**) The results for computational time when the function converges.

Following a series of trials, the optimal settings for the standard genetic algorithm (GA) parameters are identified as follows: a population size of 250, 250 iterations, a crossover rate of 0.7 and a mutation rate of 0.1 (see Figure 22a). As illustrated in Figure 22, the GA exhibits a faster convergence rate than the BSA and FA, as evidenced by the fitness function reaching convergence at the 80th iteration. Figure 22c demonstrates that the GA requires a shorter computational time than the BSA [56] and FA [57]. The GA exhibits an 8.23% enhancement in computational efficiency in comparison to the FA. Conversely, the GA exhibited a 50.17% increase in computational efficiency in comparison to the BSA.

The results of the comparative analysis of the average speed and phase offset at each related road section during different periods are presented in Table 5, Table 6 and Table 7.

**Figure 23:**
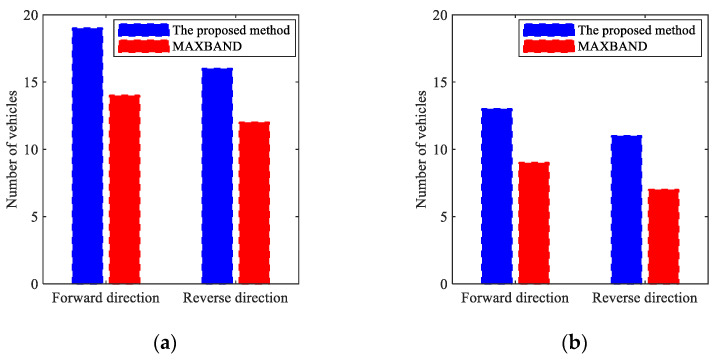
Continuously passing vehicles at the Jiangyang Middle Road Section and Wenchang West Road Section in morning peak period. (**a**) The Jiangyang Middle Road Section. (**b**) The Wenchang West Road Section.

**Figure 24:**
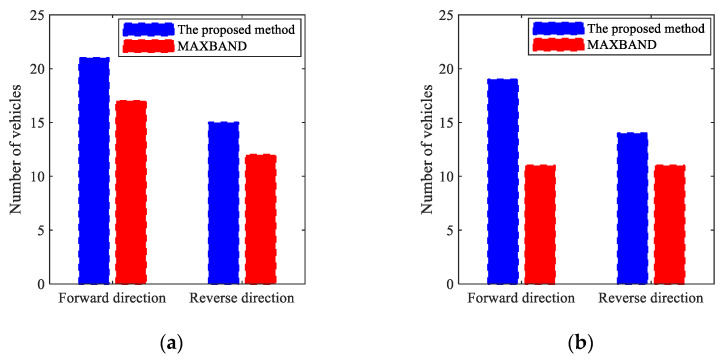
Continuously passing vehicles at the Jiangyang Middle Road Section and Wenchang West Road Section in flat peak period. (**a**) The Jiangyang Middle Road Section. (**b**) The Wenchang West Road Section.

**Figure 25:**
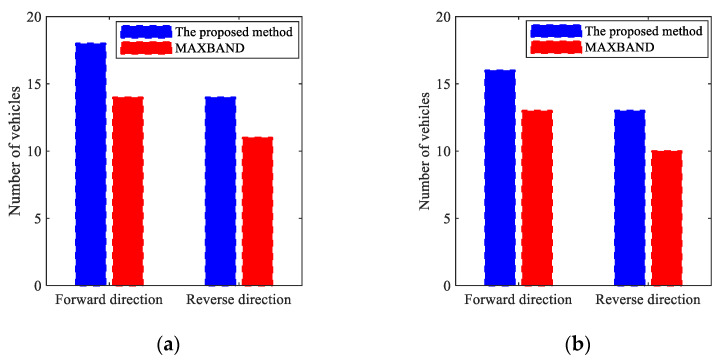
Continuously passing vehicles at the Jiangyang Middle Road Section and Wenchang West Road Section in evening peak period. (**a**) The Jiangyang Middle Road Section. (**b**) The Wenchang West Road Section.

Following the genetic algorithm (GA) optimization process, the objective function value is at its maximum when the weights of *λ_mz_* and *λ_mf_* are both 0.5, where *m* = 1, 2, 3. Figure 23, Figure 24 and Figure 25 show the comparison of the number of vehicles passing continuously through the intersection during the comprehensive green wave between the proposed method and MAXBAND.

Figure 23 demonstrates that, under the specified parameters, the application of the method results in an approximate 31.58% increase in the number of vehicles passing in the forward comprehensive green wave bandwidth during the morning peak period. Concurrently, the number of vehicles passing in the reverse comprehensive green wave bandwidth increased by approximately 35.71% during the morning peak period. In the Wenchang West Road section, the application of the method resulted in an increase of approximately 31.25% in the number of vehicles passing in the forward comprehensive green wave bandwidth during the morning peak period. Concurrently, the number of vehicles passing in the reverse comprehensive green wave bandwidth increased by approximately 41.67% during the morning peak period.

Figure 24 illustrates that the application of the method resulted in a 16.67% increase in the number of vehicles passing through the forward comprehensive green wave bandwidth in the Jiangyang middle road section during the flat peak period. Concurrently, the number of vehicles passing through the reverse comprehensive green wave bandwidth in the Jiangyang middle road section during the flat peak period was elevated by approximately 33.33%. In the Wenchang West Road section, the application of the method resulted in an increase of approximately 15.38% in the number of vehicles passing through the forward comprehensive green wave bandwidth during the flat peak period. Concurrently, the number of vehicles passing through the reverse comprehensive green wave bandwidth was elevated by approximately 27.27%.

Figure 25 shows that the implementation of the method led to a 28.57% increase in the number of vehicles passing through the forward comprehensive green wave bandwidth in the Jiangyang middle road section during the evening peak period. Concurrently, the number of vehicles passing through the reverse comprehensive green wave bandwidth increased by approximately 16.67% during the aforementioned period. In the Wenchang West Road section, the application of the method resulted in an increase of approximately 7.69% in the number of vehicles passing in the forward comprehensive green wave bandwidth during the evening peak period. Concurrently, the number of vehicles passing through the reverse comprehensive green wave bandwidth increased by approximately 22.22% during the evening peak period.

#### 4.2.3. Simulation Results

**Figure 26:**
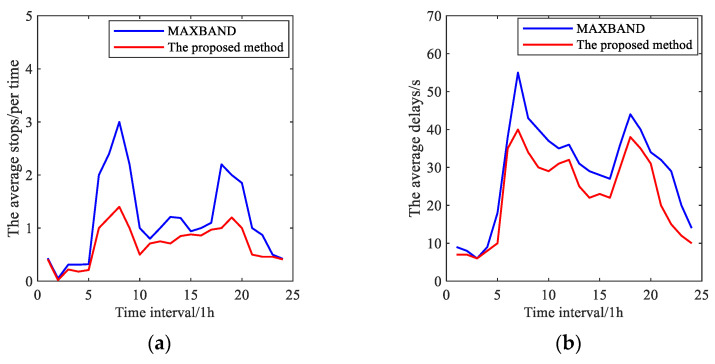
The final comparison results for average stops and delays with two control models in Jiangyang middle road section. (**a**) The average stops with two control models. (**b**) The average delays with two control models.

**Figure 27:**
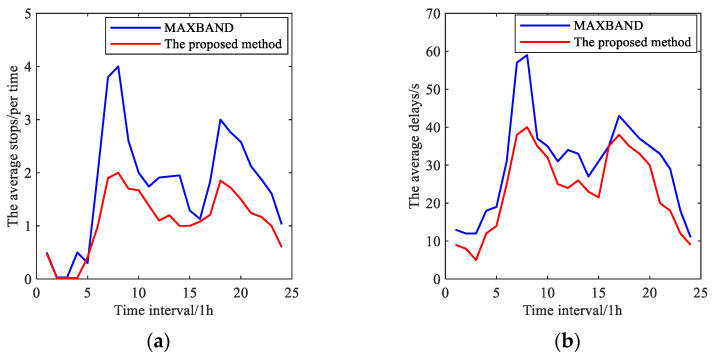
The final comparison results for average stops and delays with two control models in Wenchang west road section. (**a**) The average stops with two control models. (**b**) The average delays with two control models.

The two control schemes are simulated in accordance with the VISSIM 4.30 software. The average delays and stops are selected as the evaluation indexes of the final control effect. As illustrated in Figure 26 and Figure 27, the comparison of evaluation results reflects the average delays and stops when connected vehicles have passed through related intersection groups.

As shown in Figure 26 and Figure 27, the proposed model significantly reduces both average delays and stopping frequency across all tested scenarios. It is evident that the delays and stops are significantly reduced under the bidirectional cycle green wave coordinated control and multi-level vehicle speed guidance collaborative optimization method. The method allows for the uninterrupted passage of connected vehicles through the relevant intersection groups. The results indicate that the traffic capacity of the relevant road section and green time utilization have been enhanced as a consequence of the method.

**Figure 28:**
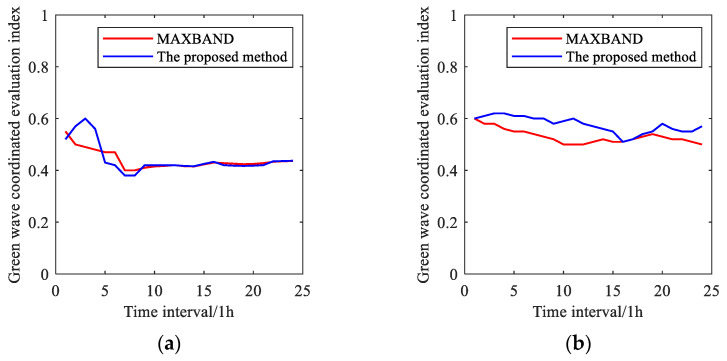

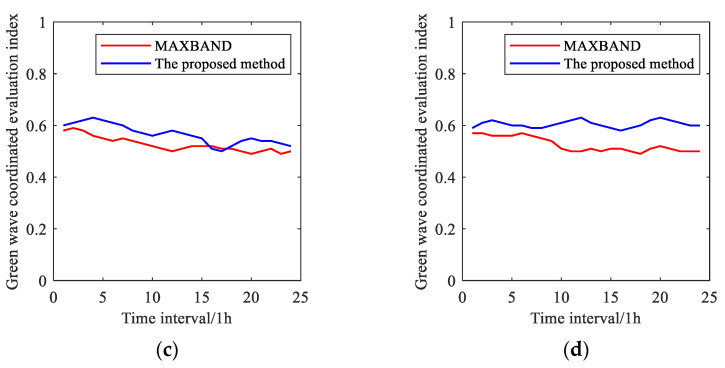
The results of the green wave coordinated evaluation index under different traffic saturation levels: (**a**) under traffic saturation level of 0.2; (**b**) under traffic saturation level of 0.4; (**c**) under traffic saturation level of 0.6; (**d**) under traffic saturation level of 0.8.

The robustness of the evaluation method is demonstrated by analysing the results of the combined green wave and turn wave under different traffic saturation levels, as illustrated in Figure 28.

Figure 28 presents the analysis of the green wave rating results under different traffic saturation levels. In summary, the proposed method consistently outperforms MAXBAND, particularly under high saturation (>0.6), due to its adaptive coordination of vehicle trajectories and phase offsets. Nevertheless, when traffic saturation is reduced to 0.2, it becomes challenging to effectively demonstrate the superiority of the method presented in this paper. This is due to the fact that the evaluation index *I_E_* for green wave coordination becomes unstable when the penetration rate is reduced, resulting in a certain degree of randomness within the arterial green wave. This instability is attributed to the greater volatility of the actual green wave traffic efficiency value *I_R_* in low permeability, which affects the final evaluation results. However, the evaluation method appears to be practically feasible. As shown in Figure 28, the proposed method outperforms MAXBAND significantly under high saturation (>0.6) due to its adaptive speed guidance and phase offset coordination. However, under low saturation (<0.3), MAXBAND exhibits comparable performance because fixed offsets suffice for sparse traffic. Future improvements could involve dynamic weight adjustment in the objective function (e.g., prioritizing bandwidth maximization during peaks and energy efficiency during off-peaks) to enhance scenario-specific adaptability.

As shown in Table 8 and Table 9, the morning peak period (7:00~8:00), flat peak period (9:00~17:00) and evening peak period (18:00~19:00) were selected for further analysis of the simulation results (including average stops and average delay) in the Jiangyang middle road section and Wenchang west road section.

Table 8 and Table 9 provide further illustration of the final evaluation indexes from the model optimization. The data presented in Table 8 and Table 9 demonstrate that the proposed model exhibits a more pronounced improvement effect than the two models, with a reduction in the average delays and stops at the intersections of the Jiangyang middle and Wenchang west road sections. In particular, during the morning peak period, the average number of stops at the Jiangyang middle and Wenchang west road sections are reduced by 53.56% and 50.00% respectively. Furthermore, the average delay experienced by vehicles travelling along the Jiangyang middle road section is reduced by 24.32% during the flat peak period. Similarly, the average delay experienced by vehicles travelling along the Wenchang west road section is reduced by 28.85% during the morning peak period. The travel times of arterial vehicles are constrained by the phase offset on a coordinated arterial. It is not possible for them to simply increase their speed in order to reduce the delay time. Consequently, the most effective method for reducing shock wave delay is to reduce the delay for arterial vehicles. In the context of the connected vehicle environment, arterial vehicles are provided with speed guidance and are permitted to pass through the stop line without stopping. As a result, the starting wave delay can be significantly reduced. At the same time, the integration of vehicle speed guidance control and coordinated phase offset optimization is employed to optimize the utilization of green time. The proposed method not only enhances the coordinated phase and optimizes the utilization of the green wave band but also facilitates the uncoordinated phase in obtaining a longer green time, thereby reducing the average delays and average stops for arterial and non-arterial vehicles.

The performance is discussed under different conditions and potential improvements, including MAXBAND, MULTIBAND (named classical green wave method), PPO (named reinforcement learning baseline) and DQNGA in Table 10. Table 10 shows that DQNGA reduces average delay by 30.19% compared to MAXBAND and 22.32% compared to MULTIBAND and 17.78% to PPO. Similarly, it can be inferred that DQNGA reduces average stops by 50% compared to MAXBAND, 39.06% compared to MULTIBAND and 30.36% compared to PPO. The results show that the proposed DQNGA model exhibits enhanced stability and precision in comparison to the other three algorithms.

## 5. Conclusions

Building upon existing research in speed guidance and green wave coordination for connected vehicle systems, current methodologies predominantly focus on primary green wave bandwidth while neglecting the potential of supplementary green waves. This study introduces an integrated optimization framework that synergizes hierarchical speed guidance with bidirectional periodic green wave coordination. The proposed methodology establishes a global optimization framework that simultaneously determines optimal guidance speeds and bidirectional green wave bandwidths through three key innovations: (1) a hierarchical speed guidance strategy minimizing travel time while maximizing intersection throughput through trajectory optimization of initial and target vehicle states; (2) a multi-objective optimization model combining maximum arterial velocity with comprehensive green wave bandwidth under connected vehicle conditions; (3) a bi-level combinatorial optimization architecture combining a Deep Q-Network (DQN) with a Genetic Algorithm (GA), termed DQNGA, which resolves complex traffic scenarios through specialized neural network configurations.

Validation via VISSIM/MATLAB co-simulation with empirical traffic data demonstrated superior computational efficiency, with DQNGA outperforming conventional MAXBAND and state-of-the-art reinforcement learning approaches (CDRL, MADQN, QT-CDQN, DTA) by 20–35% in convergence speed. Field implementation revealed substantial operational improvements: morning peak stops decreased by 53.6% and 50.0% at Jiangyang Middle Road and Wenchang West Road sections, respectively, while off-peak delays were reduced by 24.3% and 28.9% at corresponding locations. These metrics confirm the framework’s capability to enhance arterial capacity through coordinated speed–phase optimization while maintaining traffic stability.

There remain numerous research topics for future investigation, several of which are discussed in the following sections. Firstly, the follow-up study will focus on the sensitivity analysis of the transition time of vehicle acceleration and deceleration to the model. Secondly, the proposed model assumes that all vehicles are fully connected. One of the challenges in integrating traditional vehicles (mixed traffic flow) into this model is the lack of data on their behavior.

## Figures and Tables

**Table 1:** Definition of Parameters.

Symbol	Quantity
*i*	The *i*th optimization cycle
*j*	The *j*th vehicle
$V_{ij}^{end}$	The target speed of the vehicle within the control range, m/s
$V_{\max}$	Maximum permissible vehicle speed, m/s
$t_{ij}^{end}$	The target time of vehicle arrival at downstream intersection, s
$h_{ij}$	The time headway between the current vehicle and the preceding vehicle, s
$l_{ij}^{end}$	The target displacement of the vehicle within the control range, m
$h_{t}$	The time safe headway, s
$S_{0}$	The safe parking distance, m
$len_{i\left(j-1\right)}$	The front vehicle’s length of the current vehicle, m
$V_{ij}^{0}$	The initial speed of the vehicle entering the control range, m/s
$V_{i-end}^{0}$	The initial speed of the last vehicle entering the control range in the optimization cycle, m/s
$t_{ij}^{0},\;T_{ij}^{0}$	The initial time of vehicle entering the control range, s
$t_{ij}^{2},\;T_{ij}^{4}$	The time when the vehicle reaches the downstream intersection, s
$t_{ij}^{1},\;T_{ij}^{1}$	The time when the vehicle is at the end of the first uniform speed change stage, s
$T_{ij}^{2}$	The time when the vehicle ends its first uniform motion at the guiding speed, s
$T_{ij}^{3}$	The time when the vehicle is at the end of the second uniform speed change stage, s
L	The total displacement of the vehicle within the control range, m
$l_{1}$ $,\;L_{1}$	The displacement of vehicle during the first uniform speed change stage, m
$l_{2}$ $,\;L_{4}$	The displacement of the vehicle in the stage of uniform motion at *V*_max_, m
$L_{2}$	The displacement of the vehicle during the first uniform motion stage at the guiding speed, m
$L_{3}$	The displacement of the vehicle during the second uniform speed change stage, m
$V_{ij}^{1}$	The guiding speed of the vehicle entering the control range, m/s
$\Delta S\left(t\right)$	The time distance function between the current vehicle and the front vehicle
$S_{i\left(j-1\right)}\left(t\right)$	The time distance function of the front vehicle
$S_{ij}\left(t\right)$	The time distance function of the current vehicle
S	The safe space headway, m
$t_{i-end}^{0}$	The initial time when the last vehicle enters the control range in the optimization cycle, s
*P*	The control objective expression of forward green wave bandwidth.
*H*	The control objective expression of reverse green wave bandwidth.

**Table 2:** Definitions of the variables.

Variable	Definition
*tc*	The time when the *c*th gather–disperse wave intersects with the *c* − 1th gather–disperse wave at the downstream intersection
*v*_1_	The average speed corresponding to traffic flow *q_g_*_2_
*v*_2_	The average speed corresponding to traffic flow *q_r_*_1_
*v*_3_	The average speed corresponding to traffic flow *q_r_*_2_
*v*_4_	The average speed corresponding to traffic flow *q_g_*_1_
*W*_1_~*W*_5_	The gather–disperse wave speed
*q_g_*_1_	The saturation flow of queuing vehicles in coordinated phase when they release
*q_g_*_2_	The average flow of vehicles flowing out after the queue of vehicles has dissipated late in the green light of coordinated phase
*q_r_*_1_	The average flow of queuing vehicles driving out of the intersection in the pre-reddish phase of the coordinated phase interface with the non-coordinated phase
*q_r_*_2_	The average vehicle outflow after the coordinated phase red time late articulated uncoordinated phase queue has dissipated
*t_g_*_1_	The time for the vehicle queue to dissipate during the green time of coordinated phase
*t_r_*_1_	The time for completion of uncoordinated phase queue dissipation for coordinated phase articulation
*C′*	Public cycle of upstream and downstream intersections
*T_g_*	The upstream intersections with the green time of the coordinated phases
*T_r_*	The upstream intersections with the red time of the coordinated phases
*T_gb_*	Time difference between the beginning of the green wave at the upstream intersection and the beginning of the green time at the coordinated phase in the same cycle
*T_ge_*	The time difference between the end of the green wave at the upstream intersection and the start of the green light at the coordinated phase in the same cycle

**Table 3:** Some sample data.

Vehicle_ID	Global_Time	Local_X	Local_Y	Global_X	Global_Y	v	Lane_ID	Following	Space_Headway	Time_Headway
515	1,118,848,075,000	30.034	188.062	6,451,203.729	1,873,252.549	13	3	523	119.1	5.11
2224	1,113,437,421,700	41.429	472.901	6,042,814.264	2,133,542.012	14	4	2211	53.34	2.01
1033	1,118,848,324,700	6.202	1701.14	6,452,347.673	1,872,258.452	13	1	1040	38.81	0.92
744	1,118,848,181,200	28.878	490.086	6,451,422.353	1,873,041.018	15	3	752	37.8	1.54
…	…	…	…	…	…	…	…	…	…	…

**Table 4:** Definitions of Variables.

Model Parameters	Value
Replay memory *D*	40,000
The number of training times	80,000
Batch size	64
Learning rate	0.0001
Discount rate	[0.01, 0.99]
ReLU activation function	0.001
Action interval Δt/s	3

**Table 5:** The comparison results for average speed and signal offset of each section with MAXBAND and the proposed model in morning peak period.

Road Section	Intersection	Signal Offset of MAXBAND	Signal Offset of the Proposed Model	Forward and Reverse Comprehensive Green Wave Bandwidth of MAXBAND	Forward and Reverse Comprehensive Green Wave Bandwidth of the Proposed Model
Jiangyang Middle Road Section	Intersection of Jiangyang Middle Road and Huidong Road	0	15	(30, 27)	(34, 30)
	Intersection of Jiangyang Middle Road and Xingcheng East Road	126	112		
	Intersection of Jiangyang Middle Road and Yangzi River Road	77	85		
Wenchang West Road Section	Intersection of Wenchang West Road and Guozhan Road	72	77	(21, 18)	(23, 21)
	Intersection of Wenchang West Road and Ruiyang Middle Road	0	11		
	Intersection of Wenchang West Road and Baixiang Road	45	38		

**Table 6:** The comparison results for average speed and signal offset of each section with MAXBAND and the proposed model in flat peak period.

Road Section	Intersection	Signal Offset of MAXBAND	Signal Offset of the Proposed Model	Forward and Reverse Comprehensive Green Wave Bandwidth of MAXBAND	Forward and Reverse Comprehensive Green Wave Bandwidth of the Proposed Model
Jiangyang Middle Road Section	Intersection of Jiangyang Middle Road and Huidong Road	0	113	(35, 33)	(38, 35)
	Intersection of Jiangyang Middle Road and Xingcheng East Road	63	78		
	Intersection of Jiangyang Middle Road and Yangzi River Road	117	101		
Wenchang West Road Section	Intersection of Wenchang West Road and Guozhan Road	36	28	(26, 24)	(28, 27)
	Intersection of Wenchang West Road and Ruiyang Middle Road	0	119		
	Intersection of Wenchang West Road and Baixiang Road	45	52		

**Table 7:** The comparison results for average speed and signal offset of each section with MAXBAND and the proposed model in evening peak period.

Road Section	Intersection	Signal Offset of MAXBAND	Signal Offset of the Proposed Model	Forward and Reverse Comprehensive Green Wave Bandwidth of MAXBAND	Forward and Reverse Comprehensive Green Wave Bandwidth of the Proposed Model
Jiangyang Middle Road Section	Intersection of Jiangyang Middle Road and Huidong Road	0	17	(29, 26)	(32, 29)
	Intersection of Jiangyang Middle Road and Xingcheng East Road	126	118		
	Intersection of Jiangyang Middle Road and Yangzi River Road	77	91		
Wenchang West Road Section	Intersection of Wenchang West Road and Guozhan Road	72	82	(23, 20)	(26, 24)
	Intersection of Wenchang West Road and Ruiyang Middle Road	0	11		
	Intersection of Wenchang West Road and Baixiang Road	45	35		

**Table 8:** Comparison of simulation results with Jiangyang middle road section.

Road Section	Average Stops			Average Delay		
	MAXBAND	The Proposed Model	Improvement Effect	MAXBAND	The Proposed Model	Improvement Effect
Morning Peak Period	2.75	1.277	53.56%	53	43	18.87%
Flat Peak Period	1.121	0.693	38.18%	37	28	24.32%
Evening Peak Period	2.295	1.224	46.67%	47	37	21.28%

**Table 9:** Comparison of simulation results with Wenchang west road section.

Road Section	Average Stops			Average Delay		
	MAXBAND	The Proposed Model	Improvement Effect	MAXBAND	The Proposed Model	Improvement Effect
Morning Peak Period	3.9	1.95	50.00%	57	42	28.85%
Flat Peak Period	1.913	1.212	36.64%	36	30	16.67%
Evening Peak Period	3.25	1.888	41.91%	48	41	14.58%

**Table 10:** Differences in performance with different conditions and algorithms.

Road Section	Metric	MAXBAND	MULTIBAND	PPO	Proposed DQNGA
Jiangyang Middle	Average. Delay (s)	53	48	45	37
Wenchang West	Average. Stops	3.9	3.2	2.8	1.95

## Data Availability

The data presented in this study are available on request from the first author.
